# Input and Age‐Dependent Variation in Second Language Learning: A Connectionist Account

**DOI:** 10.1111/cogs.12519

**Published:** 2017-07-26

**Authors:** Marius Janciauskas, Franklin Chang

**Affiliations:** ^1^ School of Psychology The University of Liverpool

**Keywords:** Second language learning, Sensitive period, Age of acquisition, Input‐based learning, Connectionist neural network, Language acquisition

## Abstract

Language learning requires linguistic input, but several studies have found that knowledge of second language (L2) rules does not seem to improve with more language exposure (e.g., Johnson & Newport, 1989). One reason for this is that previous studies did not factor out variation due to the different rules tested. To examine this issue, we reanalyzed grammaticality judgment scores in Flege, Yeni‐Komshian, and Liu's (1999) study of L2 learners using rule‐related predictors and found that, in addition to the overall drop in performance due to a sensitive period, L2 knowledge increased with years of input. Knowledge of different grammar rules was negatively associated with input frequency of those rules. To better understand these effects, we modeled the results using a connectionist model that was trained using Korean as a first language (L1) and then English as an L2. To explain the sensitive period in L2 learning, the model's learning rate was reduced in an age‐related manner. By assigning different learning rates for syntax and lexical learning, we were able to model the difference between early and late L2 learners in input sensitivity. The model's learning mechanism allowed transfer between the L1 and L2, and this helped to explain the differences between different rules in the grammaticality judgment task. This work demonstrates that an L1 model of learning and processing can be adapted to provide an explicit account of how the input and the sensitive period interact in L2 learning.

## 1 Introduction

Linguistic input is critical for language learning. In first language (L1) acquisition, linguistic elements that occur more frequently are easier to learn (Ambridge, Kidd, Rowland, & Theakston, 2015; Bybee, 2006; Dąbrowska & Lieven, 2005; Marchman, Wulfeck, & Weismer, 1999; Phillips, 2006). However, the relationship between input frequency and second language (L2) learning is less clear. Several studies have reported that the amount of language input—as measured, for example, by years living in L2 environment—does not correlate highly with the acquisition of grammar and morphology in adult L2 learners who started learning the L2 at different ages (Andringa, 2014; DeKeyser, 2000; DeKeyser, Alfi‐Shabtay, & Ravid, 2010; Johnson & Newport, 1989; Lee & Schacter, 1997; McDonald, 2000; Oyama, 1978; Patkowski, 1980). Given that languages cannot be learned without linguistic input, these findings are counterintuitive and at odds with the notion that input plays an important role in L2 theories (N. C. Ellis, 2013; MacWhinney, 2008). This discrepancy in the role of the input suggests that differences exist in the mechanisms that are used by L1 and L2 learners, and this study examines whether these differences can be explained in a unified way.

Input effects in L2 learning are modulated by the critical or sensitive period, the time window approximately between birth and puberty during which language learning is most effective (Knudsen, 2004; Lenneberg, 1967). This effect is modulated by the age at which language learners begin learning the L2. As age of acquisition (AoA) increases, the ability to learn the L2 decreases (Flege, Yeni‐Komshian, & Liu, 1999; Johnson & Newport, 1989). While many of these AoA effects are found in explicit tasks, similar effects have been found in implicit tasks such as timed judgments (R. Ellis, 2005) and ERP studies (Weber‐Fox & Neville, 1996). Also AoA effects are found in L1 learning in deaf learners of sign language (Boudreault & Mayberry, 2006; Mayberry, 2010; Mayberry & Eichen, 1991) and international adoptees (Gardell, 1979; Gauthier & Genesee, 2011; Hyltenstam, Bylund, Abrahamsson, & Park, 2009). A wide range of social, motivational, input, and biological factors have been proposed to explain this reduction in learning ability (for a balanced review, see DeKeyser & Larson‐Hall, 2005). For these factors to explain the AoA effects, there needs to be a gradual accumulation of the negative impact of these factors as the learner gets older (e.g., motivation to learn the L2 decreases for each year of age). Understanding the mechanism that could explain the gradual reduction in L1/L2 learning in such diverse circumstances is an important goal for understanding language learning.

A classic study that investigated the sensitive period is that of Johnson and Newport (1989). The authors tested English morphosyntactic grammar knowledge in Korean and Chinese immigrants in the United States. They examined whether the English abilities of these L2 speakers could be predicted from the age at which they started learning English in immersion settings (3–39 years: *age of acquisition*, AoA), and years spend in the United States (7–30 years; *length of exposure*, LoE). The participants’ L2 knowledge was assessed via a grammaticality judgment task, in which they indicated whether a given English sentence was grammatical (1a) or not (1b).

(1a) *The farmer bought two pigs at the market*
(1b) *The farmer bought two pig at the market*

The authors found that the performance dropped as AoA increased, showing that their ability to learn grammatical knowledge depended on the age at which they started learning the L2. However, they found no correlation between LoE and grammaticality judgment scores (*r *=* *.16, *p *>* *.05) and this has been replicated in several other studies (DeKeyser et al., 2010; DeKeyser, 2000; Lee & Schacter, 1997; McDonald, 2000; cf. Flege et al., 1999). The lack of LoE effect is an important issue, as it contradicts the assumption that language ability should increase as more input is experienced (N. C. Ellis, 2013).

One reason why LoE effect was not observed in Johnson and Newport's (1989) study could be related to the variation among different rules used in test sentences. The authors examined grammatical knowledge of 12 different morphosyntactic rules (Table 1). For example, sentence (1b) violated the plural rule use that required adding –s to the plural noun “pig.” Their data suggest that as AoA increased, the average grammatical knowledge dropped at different rates for different rules. Late learners performed worse with determiners and plural rules, whereas past tense and third‐person singular rules seemed to be easier to master. Similar rule‐specific effects have also been observed in several other studies (DeKeyser, 2000; Flege et al., 1999; Johnson, 1992; McDonald, 2000). Since their analyses collapsed the data over different rules, this within‐subject variation could have obscured the effect of between‐subject factors like LoE.

**Table 1:** Examples of test items used to test the knowledge of five different grammar rules (ungrammatical rule use underlined)

Rule	Grammaticality	Example Test Item
Determiner	Grammatical	Tom is reading the book in the bathtub
	Ungrammatical	Tom is reading_book in the bathtub
Plural	Grammatical	The farmer bought two pigs at the market
	Ungrammatical	The farmer bought two pig at the market
Particle verbs	Grammatical	The horse jumped over the fence yesterday
	Ungrammatical	The horse jumped the fence over yesterday
Third‐person singular	Grammatical	Every Friday our neighbor washes her car
	Ungrammatical	Every Friday our neighbor wash her car
Past tense	Grammatical	Last night the old lady died in her sleep
	Ungrammatical	Last night the old lady die in her sleep

To understand the role that rule variation plays in sensitive period studies, we reanalyzed Flege et al. (1999) study, which was based on Johnson and Newport's (1989) original study but had a much larger sample of 240 Korean learners of English (compared to 46 participants in Johnson and Newport's study). To preview the findings, our analysis showed a significant effect of rule, which means that these learners were consistently better at judging grammaticality of some rules than others (consistent with rule differences in various L1/L2 studies; Leonard, Caselli, Bartolini, McGregor, & Sabbadini, 1992; McDonald, 2000; Mizumoto, Hayashibe, Komachi, Nagata, & Matsumoto, 2012; Rescorla & Reberts, 2002). One explanation for the rule variation is the differences in the frequency with which those rules occur in the input. Higher frequency rules are thought to yield better learning outcomes (Ambridge et al., 2015; N. C. Ellis, 2002; Lieven, 2010) and this predicts that L2 learners should be more accurate at judging the grammaticality of higher frequency rules. Another explanation is that rules that are similar across the L1 and L2 are easier to learn than those that are different (L1‐transfer/interference; Bernolet, Hartsuiker, & Pickering, 2013; Foucart & Frenck‐Mestre, 2012; Hartsuiker, Pickering, & Veltkamp, 2004; Ionin & Montrul, 2010; MacWhinney, 2005; Sabourin, Stowe, & de Haan, 2006). One challenge for transfer accounts is that there is no agreement about how to best measure L1–L2 similarity and it would be difficult to augment the Flege et al. analysis with an objective measure of L1/L2 similarity. Therefore, to contrast frequency and transfer accounts, we performed a corpus study to quantify the input frequencies for some of the rules in Flege et al.'s study and used these frequencies in the reanalysis to understand the differences in L2 learners’ performance with different rules. If the frequencies positively predicted performance in grammaticality judgment task, it would support frequency‐based approaches. If this was not the case, then that would provide indirect evidence for alternative accounts like language transfer. Finally, we used a connectionist model of L1 language acquisition to see if we could model the findings in the reanalysis to understand how input frequency and language transfer might work in L2 language acquisition.

## 2 Corpus analysis

To make a grammaticality judgment, participants read a sentence and then classify it as either grammatical or ungrammatical. One way to make this decision would be to use knowledge about the transitions between words. For example, in the sentence *The farmer bought two* *pig* *at the market,* the transition between *two* and *pig* makes the sentence ungrammatical. One way to detect this ungrammatical transition would be to test if the frequency of the bigram *two pig* was below a threshold. However, since the raw bigram frequency can differ for different words (e.g., *twenty‐three pigs* is a rare grammatical bigram), it can be hard to distinguish grammatical and ungrammatical transitions based on raw bigram frequency knowledge. An alternative statistic that automatically adjusts for this is forward conditional probability (CP), which is the raw frequency of the bigram divided by the frequency of the previous word, for example, CP = frequency of *twenty‐three pigs*/frequency of *twenty‐three*. There is a lot of evidence that CPs can explain infants’ language learning behavior (Aslin, Saffran, & Newport, 1998; Gomez & Gerken, 2000), as well as experimental results in children/adults (Jurafsky, 2003; Levy, 2008; Monaghan, Chater, & Christiansen, 2005; Thompson & Newport, 2007). Critically, there is evidence suggesting that L2 learners show a similar sensitivity to forward CPs as L1 learners in an on‐line task (Huang, Wible, & Ko, 2012). In this work, we explore whether forward CPs can explain the differences in rule performance in Flege et al.'s study. Our approach does not imply that people do not also extract other statistics such as backward CPs (e.g., frequency of *twenty‐three pigs* divided by frequency of *pigs*) or other n‐grams, and use them to aid language use (Bannard & Matthews, 2008; Chang, Lieven, & Tomasello, 2008; French, Addyman, & Mareschal, 2011; Huettig & Mani, 2016). The goal of this analysis is to provide some evidence that rule differences are related to at least one input frequency‐related measure.

To compute these statistics, we used child‐directed speech from CHILDES online child language database (MacWhinney, 2000) and adult input from a spoken subset of the Corpus of Contemporary American (COCA; Davies, 2010). From CHILDES, we used the mothers’ utterances (a total of 591,762 in 32 North American corpora; Bates, Bretherton, & Snyder, 1988; Bernstein‐Ratner, 1984; Bliss, 1988; Bloom, 1970, 1973; Bohannon & Marquis, 1977; Brent & Siskind, 2001; Brown, 1973; Clark, 1978; Demetras, 1989; Feldman & Menn, 2003; Gleason, 1980; Hall, Nagy, & Linn, 1984; Higginson, 1985; Kuczaj, 1977; Morisset, Barnard, & Booth, 1995; Ninio, Snow, Pan, & Rollins, 1994; Peters, 1987; Post, 1994; Rollins, 2003; Sachs, 1983; Soderstrom, Blossom, Foygel, & Morgan, 2008; Suppes, 1974; Valian, 1991; van Houten, 1986; Warren‐Leubecker, 1982). The remaining corpora were Cornell, MacWhinney, McCune, McMillan, Snow, and Tardif (MacWhinney, 2000). These corpora consist of the recordings of mothers talking to their children of up to 8 years of age, as well as other adults/children (e.g., investigator, father, grandparents, siblings, uncles/aunts, babysitter).

Conditional probabilities depend on rule frequencies. To compute these frequency counts, we created search terms that were based on the items used to test grammaticality in Flege et al.'s study. For example, determiner (DET) knowledge was tested with an ungrammatical sentence like *The boy is helping the man to* *build house*, which requires the knowledge that the verb *build* must be followed by a determiner *the* before using the noun *house*. Thus to judge the grammaticality of the sentence, participants could use their knowledge about how likely a verb is followed by a determiner. To calculate this, we extracted the frequency of verbs followed by determiners (verb‐determiner) and the overall frequency of verbs (verb frequency) using the corpora tiers that were coded for syntactic categories and morphology. The DET rule CP was then calculated by dividing verb‐determiner frequency by the verb frequency and this tells us out of all verb uses in this corpus, what proportion were followed by a determiner. In addition to the determiner rule, we also collected CPs for four other rules: plural (PL), particle use in phrasal verbs (PAR), third‐person singular verb inflection (3PS), and past tense (PST). The PL CP was calculated by dividing the number of plural nouns by the total number of nouns, which provided a measure of how likely a plural rule was to be encountered in the input compared to other noun forms. The PAR CP was thus calculated by taking the frequency of verbs followed directly by a particle and dividing it by the total number of verbs, and this probabilistic knowledge could help to identify non‐adjacent particles as ungrammatical (e.g., *The man climbed the ladder up carefully*). The 3PS CP was calculated by dividing the number of verbs in third‐person singular form by the total number of verbs, and this could help identify how likely a 3PS form was to be encountered. The PST CP was calculated by dividing the number of past tense verbs by the total number of verbs, and this provides information about how likely past tense was in general. Table 2 shows the implemented CLAN search terms (MacWhinney, 2000) and the corresponding raw frequency for each rule (number of utterances that matched).

**Table 2:** Corpora search terms and raw frequency for different rules (the words that correspond to the search term are underlined in the example sentences)

Rule	Search Term (Example Utterances)	Raw Frequency
DET	+tMOT +t%mor +u +sdet\|* (see if we can build a tower)	159,107
PL	+tMOT +t%mor +u +sn\|*‐PL (that's what the chickens say)	40,171
PAR	+tMOT +t%mor +u +sadv\|* (you can sit some people down here)	133,958
3PS	+tMOT +t%mor +u +sv\|*‐3S (the square goes in the square)	16,570
PST	+tMOT +t%mor +u +sv\|*‐PAST (look what happened here)	6,049
VERBDET	+tMOT +t%mor +u +sv\|*^det\|* (see if we can build a tower)	42,038
VERBPAR	+tMOT +t%mor +u +sv\|*^adv\|* (go ahead)	28,571
VERB	+tMOT +t%mor +u +sv\|* (look at that)	334,191
NOUN	+tMOT +t%mor +u +sn\\|* (it's a chicken)	320,650

Table 3 shows rule conditional probabilities for the same rules. It also includes rule CPs extracted from a subset of the COCA corpus to show that the results are consistent across different corpora. The correlation between rule CPs in the CHILDES and COCA corpora was high (*r *=* *.74), which means that the frequency of these five rules was similar across both children‐ and adult‐directed speech. This correlation is due to the fact that the CPs for the DET/PL rules are higher than the 3PS/PST rules in both corpora, but the rank order within these rules is not always consistent. Since the COCA corpus was a transcription of television news programs (e.g., discussions of the Peacemaker missile system), we view this as being less typical of the input that L2 learners are generally exposed to in day‐to‐day settings. Since the CHILDES corpora include conversational speech between adults and other adults, as well as children of up to 8 years of age, we view them as a better measure of the frequent word and structures that L2 learners are likely to use and know, and hence the following analyses used the rule CPs from the CHILDES corpora only.

**Table 3:** Rule conditional probability (CP) in CHILDES and Corpus of Contemporary American (COCA) corpora

Rule	Formula Used to Calculate Rule CP	CHILDES CP	COCA CP
DET	VERBDET/VERB	0.126	0.14
PL	PL/NOUN	0.125	0.21
PAR	VERBPAR/VERB	0.085	0.13
3PS	3PS/VERB	0.05	0.08
PST	PST/VERB	0.018	0.11

This corpus analysis has provided two measures of frequency for each rule: raw frequency and CP. In the next section, we will test these different measures to see which best explains the rule differences in the Flege et al. study. If there is a significant positive effect of either frequency measure, then that would suggest that the 240 participants in that study had better knowledge of rules that were frequent in the input.

## 3 Flege et al. (1999) reanalysis

Flege and his colleagues investigated the knowledge of English grammar in 240 Korean immigrants living in the United States who had migrated at the ages between 1 and 23 (*M *= 12, *SD *= 5.9). At the time of testing, their average age ranged from 17 to 47 (*M *= 26, *SD *= 6). All participants had lived in the United States from 7 to 30 years (*M *= 14.6, *SD *= 4.6). Half of the participants were males or females and different AoA groups had representative sample of participants with different LoE (Table 4).

**Table 4:** Number of participants in different age of acquisition (AoA) and length of exposure (LoE) groups

LoE Groups	AoA Groups			
	1–5	6–11	12–17	18–22
7–14	6	35	54	32
15–30	42	37	18	16

The authors tested morphosyntactic knowledge for 10 rules using a grammaticality judgment test consisting of 144 sentences. The items were designed so that each grammatical sentence had an ungrammatical counterpart that violated a certain grammar rule (see Table 1 for examples). The participants heard a recorded sentence and were required to indicate if it was permissible in the English language. Consistent with Johnson and Newport's (1989) results, Flege et al. (1999) found that the scores for different rules varied with AoA, but their analysis involved separate anova models for each rule. The novel feature of our reanalysis is to include rule‐related predictors in the model to factor out rule variation from individual variation in LoE and AoA. In addition, we used logistic mixed effects models that could predict binary grammatical judgments for individual sentences while factoring out participant and test item variation. Since our goal was to examine how input variation influenced the acquisition of different L2 rules, we excluded the data from native English speaker and only used the data from the five rules (DET, PL, PAR, 3PS, PST) for which we had objective and comparable search terms. Since grammatical sentences must conform to multiple grammatical rules, we used the ungrammatical test items, because correct rejection of these rules is more likely to relate to the rule that was used to make the sentence ungrammatical. There were eight test sentences for each rule (except for PAR which only had 6 items) and overall there were 9,120 judgments for the 38 test items over 240 participants.

**Figure 1:**
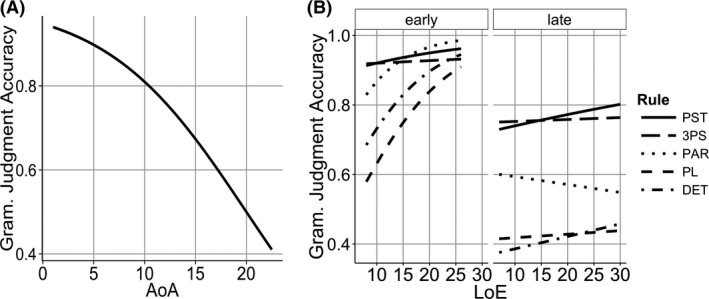
(A) Effect of age of acquisition (AoA) on grammaticality judgment scores. (B) Effect of length of exposure (LoE) on different rules in early (<12) and late (>12) AoA learner.

To replicate the earlier studies that found no effect of LoE, we first analyzed the data without including any rule‐related predictors. Grammaticality judgments (grammatical* *= 1, ungrammatical* *= 0) were predicted by a logistic mixed model with AoA crossed with LoE (all predictor variables were centered) and participant and test sentences as random effects. The maximal model that converged contained AoA crossed with LoE as random slopes for test sentence (R version 3.0.2; Barr, Levy, Scheepers, & Tily, 2013). Likelihood‐ratio tests were used to compare models and a chi‐squared statistic for the comparison was used to compute *p*‐values. The same approach was used for all the models in this paper. As seen in Fig. 1A, there was a significant effect of AoA which suggests an age‐related reduction in L2 learning ability (β = −0.2, *SE *= 0.02, χ^2^(1)* *= 65.98, *p* < .001). There was no effect of LoE (*p* = .17) and no interaction between the two variables (*p* = .25). Thus, we find that the years of input is not a strong predictor of grammaticality judgments when the variability between rules is treated as unexplained variance.

Next, we added rule as a categorical factor (fully crossed with AoA and LoE) to see if L2 learners showed consistent patterns in their knowledge for certain rules. The maximal model that converged contained no random slopes. There was a significant negative effect of AoA (β* *= −0.161, *SE *= 0.02, χ^2^(1) * *= 177.51, *p* < .001), a positive effect of LoE (β* *= −0.001, *SE *= 0.03, χ^2^(1)* *= 4.13, *p* = .042), and a negative effect of rule (χ^2^(1)* *= 24.28, *p* < .001). There was a marginal interaction between AoA and LoE (β* *= 0.003, *SE *= 0, χ^2^(1)* *= 3.08, *p* = .079). There was also a significant interaction between AoA and rule (χ^2^(1)=61.78, *p* < .001). Finally, there was a three‐way interaction between AoA, LoE, and rule (χ^2^(1)* *= 13.56, *p* = .0088). This analysis demonstrates that participants with different AoA and LoE show consistent differences between the rules that they are tested on (e.g., judgments of past tense rule items were consistently better than judgments of determiner rule items). When this rule‐related variability was factored out, then LoE showed a significant positive effect, where more years of input led to better knowledge of English grammar. Thus, the weak nature of LoE effects in previous studies could be due to the fact that earlier analyses treated rule variation as unexplained variance. The variation due to rule can be clearly seen in Fig. 1B, where we split AoA into early learners (<12 years) and late learners (>12 years, both 120 participants). We used 12 years, because this is where a non‐linearity occurs in the data (Flege et al., 1999), but we make no claim about the special role of this particular age.

The above analysis suggests that there are consistent differences among the rules, but since rule is a factor, each level of rule is treated as an arbitrary category and the analysis provides no explanation for these rule differences. One possible explanation of these rule differences is that participants rely on the knowledge of the raw frequency of the categories at the critical point in the test utterances. For example, knowing how frequently a verb is followed by a preposition can help to identify the error in the PAR rule item *The horse* *jumped* *the fence over yesterday*. To test this hypothesis, we tested a fully crossed model with categorical rule replaced by centered frequency for the adjacent categories at the critical point. The maximal model that converged contained random slope of AoA for test sentence and no slopes for participant. There was a significant negative effect of AoA (β = −0.2, *SE *= 0.02, χ^2^(1)* *= 68.8, *p* < .001), a marginal effect of LoE (β = 0.05, *SE *= 0.02, χ^2^(1)* *= 3.7, *p* = .055), and a negative effect of frequency (β = −0.00001, *SE *= 0.000003, χ^2^(1)* *= 4.96, *p* < .03). There was a marginal interaction between AoA and LoE (β = −0.006, *SE *= 0.004, χ^2^(1)* *= 2.99, *p* = .08). There was also an interaction between AoA and frequency (β = −0.0000005, *SE *= −0.0000002, χ^2^(1)* *= 6.29, *p* = .012). Finally, there was a three‐way interaction between AoA, LoE, and frequency (β = −0.00000005, *SE *= 0.00000003, χ^2^(1)* *= 3.84, *p* = .05). This analysis suggests that the rule differences in judgment behavior can be explained by a frequency measure. But unlike the previous model with rule as a factor, this model found only a marginal effect of LoE. Furthermore, since raw frequency will vary with the frequency of the component categories and the size of the corpus, we will test whether forward CPs, which are less sensitive to these factors, can explain this rule variation.

The next model included forward rule CP fully crossed with AoA and LoE. Rule CPs are computed from the raw frequencies divided by the previous category and hence they can vary between 0 and 1 (regardless of the frequency of the corresponding categories or the corpus size). The maximal model that converged contained random slopes for rule CP for participants and random slopes for AoA for test sentence. There was a significant negative effect of AoA (β = −0.2, *SE *= 0.02, χ^2^(1)* *= 68.8, *p* < .001), a positive effect of LoE (β = 0.04, *SE *= 0.02, χ^2^(1)* *= 4.17, *p* = .04), and a negative effect of rule CP (β = −21.3, *SE *= 3.76, χ^2^(1)* *= 20.1, *p* < .001). There was a significant interaction between AoA and LoE (β = −0.01, *SE *= 0.004, χ^2^(1)* *= 4.94, *p* = .03). There was also a marginal interaction between AoA and rule CP (β = −0.5, *SE *= 0.33, χ^2^(1)* *= 3.37, *p* = .07). Finally, there was a three‐way interaction between AoA, LoE, and rule CP (β = −0.1, *SE *= 0.04, χ^2^(1)* *= 5.93, *p* = .015). This shows that as AoA increased, the weakening effect of LoE affected higher CP rules more that lower CP rules.

One puzzle in the L2 literature is that years of studying an L2 do not seem to positively predict knowledge of the L2 (DeKeyser, 2000; DeKeyser et al., 2010; Johnson & Newport, 1989; Lee & Schacter, 1997; McDonald, 2000). We replicated this finding (non‐significant LoE) in our first model without any rule‐related predictors. Furthermore, a model that included raw frequency did not yield a significant effect of LoE, suggesting that this predictor did not factor out rule variations sufficiently to be able to see the effects of LoE. But when rule was added as a factor or as rule CP, we found a significant positive effect of LoE, where performance improved with more linguistic exposure. In addition, while all the models exhibited a sensitive period effect (a reduction in grammatical knowledge with increased AoA), only the rule CP model exhibited a significant interaction between LoE and AoA, where late learners benefitted from the input less than early learners. We suggest that previous studies did not find positive effects of LoE or interactions of LoE with other factors, because they did not fully factor out variation between rules.

In addition to clarifying the effect of AoA and LoE, these rule‐related predictors in the model suggested that some rules were consistently easier than other rules, regardless of the test sentence they were in or participant differences. Both the raw frequency and rule CP models suggest that these rule differences are due to a negative relationship with frequency. This conflicts with theories of L1 and L2 learning which argue that higher frequency should lead to greater accuracy (Ambridge et al., 2015; N. C. Ellis, 2002) and this work will attempt to explain this discrepancy. To better understand this negative effect, we need to determine which measure of frequency provides the best account of the data. One way to compare these models is with *R* ^2^, which is the variance explained by each model (Johnson, 2014; Nakagawa & Schielzeth, 2013). The model without rule CP explained about 21% of the variance. The model with raw frequency explained an extra 4% (*R* ^2^ = .25) and the rule CP model explained about 9% more (*R* ^2^ = .30). Since the rule CP model explained the most variance and uses a measure of frequency that is less dependent on word and corpus properties, we will use rule CP as our proxy for frequency in L2 learning.

The rule CP model revealed a significant three‐way interaction between AoA, LoE, and CPs. This indicates that the weaker effect of LoE in later AoA learners impacted higher CP rules more than lower CP rules. Specifically, Fig. 1B shows that the high CP rules DET and PL have a strong positive LoE slope in early AoA learners, but the slope is smaller in late learners. However, the slopes of lower CP rules like PST and 3PS were less affected by AoA. This suggests that late AoA learners have trouble using the high frequency of higher CP rules to acquire them better.

In sum, our reanalysis of Flege et al.'s data suggested a complex set of mechanisms in L2 grammatical learning. These learners showed a sensitive period effect (negative effect of AoA). In support of frequency‐based approaches (e.g., N. C. Ellis, 2002), we found that the amount of input (LoE) had a positive effect on L2 learning, but this was reduced in late learners. However, frequency‐based approaches cannot explain the negative effect of rule CP, where frequent noun‐based rules were associated with lower accuracy scores than less frequent verb‐based rules. Since each of the 240 participants was tested on each rule, the difference in the rules cannot be easily attributed to between‐participant differences in motivation, social factors, or biological factors. A likely cause of the rule differences is transfer from L1, since Korean does not have determiners and uses plural marking less than English. Support for the transfer account can be found in Ionin and Montrul (2010), who found that Korean learners of English had more trouble learning the generic interpretation of English determiners compared to matched Spanish learners, and this is presumably because Spanish speakers could use determiners in their L1 to enhance their learning of English. However, the Korean learners also learned third‐person singular verbs fairly easily even though the Korean language does not mark this distinction, so it is not obvious what kind of transfer mechanism could explain the learning of this rule. One possible account of language transfer are connectionist learning mechanisms that can encode similarity structure using distributed representations (Twomey, Chang, & Ambridge, 2014). In the next section, we examine whether a connectionist model is able to explain the findings in our reanalysis.

## 4 A connectionist model of the acquisition of morphosyntactic rules in L2

In the present work, we developed a computational model of L2 language acquisition and sentence processing and used it to examine the results observed in our Flege et al. reanalysis. The model is based on the connectionist model of L1 learning and processing called the dual‐path model (Chang, 2002). The model has several features that are relevant for its application to this dataset. First of all, the model has been shown to be able to learn abstract English grammatical constraints like those that are tested in Flege et al.'s study (Chang, Dell, & Bock, 2006). Second, the model can learn typologically different languages (Chang, Baumann, Pappert, & Fitz, 2015) and, in particular, it has been shown to be able to learn and explain various Japanese phenomena (Chang, 2009), which is a verb‐final case‐marked language like Korean. Finally the model uses linguistic input to make small changes to its morphosyntactic knowledge within a limited capacity memory and this means that the knowledge that it learns for different rules may compete with or support learning of new rules (Fitz, Chang, & Christiansen, 2011; Twomey et al., 2014).

To simulate the environment of L2 learning at different ages, we first trained the dual‐path model on Korean‐like L1 input until it reached adult‐like performance. The weights in the Korean model were saved after every 3,000 epochs (1,000 epochs represented one human year) and were used as the starting points for the models learning English as an L2. By varying the starting point, we simulated children who had different amounts of Korean knowledge before moving to an English‐speaking environment at different ages (AoA). Since the same model weights are used to learn both languages, the model instantiates the idea that shared systems are used for both L1 and L2 languages (Hartsuiker & Pickering, 2008; Hartsuiker et al., 2004; Schoonbaert, Hartsuiker, & Pickering, 2007). This shared system assumption combined with the model's learning mechanism is consistent with evidence for transfer between L1 and L2 in various tasks (e.g., structural priming; Chang et al., 2006).

### 4.1 The Korean L1 and English L2 input environment for the models

Both the Korean and English languages consisted of simple intransitive, transitive, and dative structure sentences. The languages were composed of 40 words: eight animate nouns, eight inanimate nouns, six transitive, six intransitive, and six dative verbs. The Korean language included function words/morphemes (particles) that denoted case (e.g., nominative *ka,* accusative *ul,* dative *ey key*) and verb endings (e.g., *‐da*). The English language contained morphemes to mark tense *(‐ed, ‐ing*), third‐person singular verb inflection (*‐ss*), noun number (*‐z*, this letter was chosen to differentiate it from third‐person singular inflection), and determiners (*a, an, the, this, that, two, three, many, several*) with the appropriate plural counterparts. To test particle movement rules, the grammar also contained two prepositions for creating phrasal verbs *(down, up*).

To train the models, sentences were paired with corresponding messages. Intransitive sentences had one argument Y in the message that mapped onto the subject slot. Transitives had an agent X and a patient Y argument that mapped onto the subject and object slots, respectively. Finally, datives had an agent X, a patient Y, and a goal Z argument that mapped onto the subject, object, and indirect object slots (Table 5). Each argument was made up of a concept (e.g., CAT) and features that helped to structure the noun phrase (e.g., Y = CAT, THREE,DIST). There was a special argument for lexical action information (e.g., A = DANCE). In addition, the message contained event‐semantics (e.g., E = PROG,YY), which had information about tense and aspect of the event. There were two possible tenses (present, PAST) with two possible aspects (simple, PROGressive). Present tense and simple aspect were considered default and had no event‐semantic features. The event‐semantics also contained features that encoded the number of roles that were required to describe a given event (XX, YY, ZZ). Both Korean and English languages shared the same meaning system but used different words in the lexicon to express the message. For simplicity, the Korean content word vocabulary was created by adding the letter “k” to the beginning of the English content words (the labels play no role in the model's behavior).

**Table 5:** Examples of sentence structures used to train the model and the message that denoted the role of each constituent in the sentence

Structure	English/Korean Sentences	Message
Intransitive	those cat ‐z are dance ‐ing kthat kcat ka ksit ‐iss ‐da	A = DANCE Y = CAT, THREE, DIST E = PROG, YY
Transitive	the cat was carrying ‐ing this apple kcat ka kthis kapple ul kcarry ‐iss –eoss ‐da	A = CARRY X = CAT Y = APPLE, PROX E = PAST, PROG, XX, YY
Dative	an elk give ‐ss sugar to the cat kelk ka kcat eykey ksugar ul kgive –da	A = GIVE X = ELK, INDEF Y = SUGAR, PLUR, PROX Z = CAT E = XX, YY, ZZ

The language had features that captured some of the constraints in different rules in English and Korean (Table 6). Each noun argument in the message had a *kind* feature and a *number* feature that helped create noun phrases. The kind feature could be DEFinite, INDEFinite, PROXimate, or DISTal. The number feature could be SINGular, TWO, THREE, PLURal. All kind features were equally frequent and the singular feature was eight times more frequent than other number features. If the argument had PLUR number feature, then the noun was followed by *–z* (plural morpheme). PLUR nouns were preceded by the word *those* if the kind feature was DIST, the word *these* if the kind feature was PROX, the number word (e.g., *two*) if the kind feature was DEF, the word *the* if the number feature was PLUR, and nothing if the kind feature was INDEF. If the number feature was SING, then DEF mapped to the word *the*, INDEF mapped to the word *a*, PROX mapped to the word *this*, and DIST mapped to the word *that*. If the kind feature was INDEF, then the TWO number feature mapped to the word *several* and the THREE number feature mapped to the word *many* (otherwise TWO mapped to the word *two* and THREE mapped to the word *three*). If the kind feature was INDEF and number was SING and the following noun started with a vowel, then the article *a* was changed to the word *an*. If the noun was a liquid or mass noun like *sugar*,* milk*,* water*, or *coffee* in the plural form, then the article was omitted. In the Korean language, there were no articles except for *kthis* and *kthat*, which were signaled by the PROX and DIST features. Number features like TWO mapped to *ktwo* and THREE mapped to *kthree* in prenominal position, but there was no other plural marking. The complex nature of English noun phrase rules is one possible reason that Korean learners of English have trouble judging the grammaticality of DET and PL rules.

**Table 6:** Language constraints in English and Korean

Relevant Rule	Relevant Message Features	English	Korean
DET, PL	X = DOG, DEF, SING	*the dog*	*kdog*
DET, PL	X = DOG, INDEF, SING	*a dog*	*kdog*
DET, PL	X = DOG, PROXIMATE, SING	*this dog*	*kthis kdog*
DET, PL	X = DOG, DISTAL, SING	*that dog*	*kthat kdog*
DET, PL	X = DOG, DEF, TWO	*two dog –z*	*ktwo kdog*
DET, PL	X = DOG, INDEF, TWO	*several dog –z*	*ktwo kdog*
DET, PL	X = DOG, INDEF, THREE	*many dog –z*	*kthree kdog*
DET, PL	X = DOG, PROXIMATE, TWO	*these dog –z*	*kthis kdog*
DET, PL	X = DOG, DISTAL, THREE	*those dog –z*	*kthat kdog*
DET, PL	X = DOG, DEF, PLUR	*the dog ‐z*	*kdog*
DET, PL	X = DOG, INDEF, PLUR	*dog –z*	*kdog*
DET, PL	X = DOG, PROXIMATE, PLUR	*these dog ‐z*	*kthis kdog*
DET, PL	X = DOG, DISTAL, PLUR	*those dog ‐z*	*kthat kdog*
PAR	A = TURNDOWN E = PAST, SIMP	*turn –ed down*	*kturndown –eoss –da*
3PS	A = TURN E = PRES, SIMP	*turn ‐ss*	*kturn ‐da*
PST	A = TURN E = PAST, SIMP	*turn ‐ed*	*kturn –eoss ‐da*
	A = TURN E = PRES, PROG	*is turn ‐ing*	*kturn –iss ‐da*
	A = TURN E = PRES, PROG	*was turn ‐ing*	*kturn –iss –eoss ‐da*

There were also rules for verb construction that depended on the event‐semantic features. If the features had PROG, then the verb was followed by *–ing* and preceded by the word *is* if the feature PRES was active or the word *was* if the feature PAST was active. If the aspect was simple, then *–ed* was added after the verb for the PAST feature or *–ss* for the PRES feature. If the subject was plural, then the word *is* was changed to the word *are*, the word *was* was changed to the word *were*, and the *–ss* marking was removed. In Korean, simple PRES verbs were followed by *–da*, simple PAST verbs by *–eoss –da*, PROG PRES verbs by *–iss –da*, and PROG PAST verbs by *–iss –eoss ‐da*. In English, there were several phrasal verbs. There were intransitive verbs *give‐up* and *show‐up* that combined dative verbs *give* and *show* with the prepositions *up*. There were two transitive verbs *turn‐down* and *break‐down* that combined intransitive verbs *turn* and *break* with the preposition *down*. In Korean, these phrasal verbs were treated as separate verb forms. Therefore, the Korean model will have to learn that in English, verbs like *turn* can have two forms with different syntactic constraints and this should complicate the learning of the PAR rule. Although English and Korean have different rules for verbs, they are less different from each other in this respect.

The grammar was created to match the order in which the five rules occurred in the corpus analysis in terms of their CPs (Table 7). The CPs for these rules in the model's training set were extracted using the same formula as in the corpus analysis. Since the language was a simplified version of English, the model input CP values only match the relative order of CPs in the human data (correlation between the two is .95).

**Table 7:** Rule conditional probabilities (CPs) in English corpora and in the grammar of the model

Rule	Corpora Rule CP	Model Rule CP
DET	0.126	0.47
PL	0.125	0.4
PAR	0.085	0.22
3PS	0.05	0.16
PST	0.018	0.11

To train the models, 10 randomly generated training sets of 20,000 message‐sentence pairs were created for each age of L2 acquisition. This created 10 model subjects for each different AoA group. The message was excluded from 25% of the training pairs to increase the syntactic nature of the learned representations.

### 4.2 Dual‐path architecture

**Figure 2:**
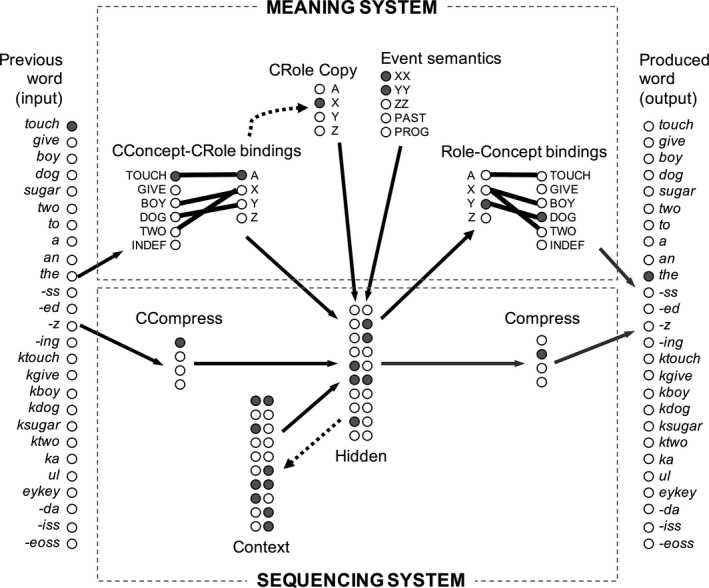
Dual‐path architecture. Black/gray arrows represent connections that have to be learned via back‐propagation of error. Thick lines represent fast‐changing message weights. Dotted arrows show copy links.

The dual‐path architecture is a connectionist architecture that can learn abstract rule‐like syntactic representations that interact with messages in sentence production (Chang, 2002). It has two pathways; sequencing pathway for learning sentence structure (lower half of Fig. 2) and meaning pathway for learning word to role mappings (upper half of Fig. 2). To adapt the model for L2 learning, the input and output layers have word units for the words in both English and Korean languages. Otherwise, the other features of the model are similar to the previous L1 versions of the dual‐path model.

The sequencing pathway is based on a simple recurrent network (SRN) architecture (Elman, 1993). The network attempts to predict the next word in a sequence from the previously heard word. The previous word is an activation pattern in the Previous Word (Input) layer. Activation spreads from the Previous Word layer to the Hidden layer via a CCompress layer and then from the Hidden layer to the Produced Word layer via another Compress layer. The function of the two compress layers is to force the model to form grammatical categories instead of learning individual word‐to‐word mappings (Elman, 1993). The Hidden layer learns and stores representations (activation patterns) that maps between the categories of the previous word and the next word and it also receives input from a Context layer that holds a copy of the Hidden layer's activation at the previous time step (dotted arrows in Fig. 2). This allows the model to learn longer distance dependencies between elements (Christiansen & Chater, 1999b).

The model learns through back‐propagation of error (Rumelhart, Hinton, & Williams, 1986). At the beginning of the training, the weights are initialized randomly with a range of 0.5. First, activation spreads through the network and generates a prediction about the next word in a sentence. The mismatch between the predicted Produced Word activations and the target is called *error*, and it is used to make small changes in the connection weights that generated the prediction. This error signal is then propagated back through the network adjusting the connection weights between all layers so that the predicted output better matches the target. Using this mechanism, the model learns weights that encode the structure of the language (all solid arrows in Fig. 2).

The sequencing system interacts with the message information in the meaning system. The message is instantiated in weights between a set of Role units and the Concept layer (Role‐Concept bindings). When the message contains Y = DOG, the Y role unit is linked to the concept DOG with a weight of 6 (thick black lines in Fig. 2). Since the Concept layer is linked to the Produced Word layer, the model can learn to activate a particular word when the appropriate concept is activated (concept DOG would activate *kdog* in Korean and *dog* in English). To allow the sequencing system to know which roles are present in the message, the Event Semantics layer has units that signal the number of roles. For example, if this layer had XX and YY units activated, that would signal to the sequencing system that it should activate the agent X Role unit after the first determiner (since English agents tend to occur early in sentences). In contrast, the Korean model would learn to activate the agent X role in sentence initial position and would also learn to activate the subject particle *ka* afterward to mark its role. In addition, the meaning system has a comprehension message, which tells the model the role of the previous word in the sentence, which helps the model produce structural alternations (e.g., active/passive). This system maps the Previous Word layer to the CConcept layer, which is linked to the CRole layer with a reverse copy of the Role‐Concept links (thick black lines on left side of Fig. 2). There is also a CRole Copy layer that helps the model keep track of the roles that have been processed.

In the present work, we apply the dual‐path model to explain L2 behavioral data in the Korean L2 English learners in the Flege et al. study. In the present work, we train models using Korean language as an L1 and then expose them to English as an L2. Consistent with the claim that L1 and L2 involved the same learning mechanism, we have kept the L2 version of the dual‐path model as similar as possible in its architecture and parameters to L1 English versions of the model (e.g., Twomey et al., 2014).

### 4.3 Evaluating the model's English grammatical knowledge

To gauge the overall learning of the language at different AoAs in the 10 models, we assessed the word prediction accuracy every 3,000 epochs using 200 randomly generated test sentences. To see how successfully the model learned the grammatical constraints in the rules in the Flege et al. study, we also examined its ability to distinguish grammatical and ungrammatical versions of the five rules in our reanalysis (DET, PL, PAR, 3PS, PST). Each test item had a matched grammatical and ungrammatical version (Table 8), and there were 100 items for each of the five rules.

**Table 8:** Grammatical and ungrammatical sentences used to test models’ performance with different rules

Rule	Error Type	Example
DET	Grammatical	*A boy touch –ed the apple*
	Determiner omission	*A boy touch –ed_apple*
PL	Grammatical	*Two boy –z touch ‐ed the apple*
	‐z morpheme omission	*Two boy_touch ‐ed the apple*
PAR	Grammatical	*A boy break ‐ss down the apple*
	Particle omission	*A boy break –ss __ the apple*
3PS	Grammatical	*A boy touch –ss the apple*
	‐ss morpheme omission	*A boy touch _**_** the apple*
PST	Grammatical	*A boy touch –ed the apple*
	‐ed morpheme omission	*A boy touch __ the apple*

To test the model's knowledge of each rule, sum of squares prediction error (the difference between the actual activation and the target activation for the word layer) for the target word at the part of the sentence where the grammatical and ungrammatical sentences differed was computed for both versions. For example, to test DET rule in the sentence *a boy touch –ed the apple*, the error of predicting the article *the* was compared to the error of predicting the word *apple* when the article was omitted as in *a boy touch –ed apple*. For each rule, the average sum of squares error (SSE) was calculated for both the grammatical and ungrammatical items. Then a rule proportion measure was computed by dividing the average SSE of ungrammatical sentences by the sum of the average SSEs for both grammatical and ungrammatical sentences. Since error levels should be larger for ungrammatical sentences than grammatical sentences, higher rule proportion scores express better rule knowledge. If the model has not developed strong expectations about whether the verbs tend to be followed by determiners or not, then SSEs for both should be similar and rule proportion should be close to 0.5. Rule proportion in the simulations approximated the grammatical judgment accuracy measure in the Flege et al.'s study and our goal is to see if the model shows similar results to those observed in the reanalysis of their data. It is known that in ERP studies (e.g., Weber‐Fox & Neville, 1996), the brains of L2 learners generate mismatch signals and this means that there is evidence that implicit prediction error signals like SSE are generated in their brains and could be used to make grammaticality judgments. However, since L2 tasks vary in their dependence on implicit and explicit knowledge (R. Ellis, 2004, 2005, 2006), different tasks might have different assumptions about the way that implicit signals like SSE are used to make behavioral choices.

### 4.4 Model simulations

We present several different simulations that attempt to approximate the L2 results in the Flege et al.'s reanalysis. Our first simulation tested whether the model's activation function could create the age‐dependent sensitive period. The second simulation manipulated the sensitive period by reducing the model's learning rate after puberty. The third simulation introduced different learning rates for the lexical and syntactic parts of the model. Finally, the fourth simulation implemented a model that received both English and Korean input to mimic the learning environment of many L2 learners.

#### 4.4.1 Simulation 1: Activation function‐based sensitive period effects

The activation function that is typically used in back‐propagation has been argued to create sensitive period effects (Elman, 1993; A. W. Ellis & Lambon Ralph, 2000; Marchman, 1993; Mermillod, Bonin, Méot, Ferrand, & Paindavoine, 2012; Munakata & McClelland, 2003; Zevin & Seidenberg, 2002). In these models, activation is spread forward in the network and the net input for a unit is the weighted sum of input activations. The net activation is passed through a logistic/sigmoid activation function to create the output activation. When the weighted sum input is 0, the logistic output activation will be 0.5. On the backward pass, the output activation is compared to the target to compute the error and this error is back‐propagated through the network to change the weights. The first step of this back‐propagation involves the computation of the derivative of the activation function. For the logistic activation function, the derivative is highest when the output activation is near 0.5 (derivative = *o* (1−*o*) when *o* is the output activation). The derivative of the activation function modulates the effect of error so that the same amount of error will have a larger effect on the weights when the weighted sum input is close to 0. When the weights are small, the weighted sum input to a unit will be small and the large derivative will allow relatively large weight changes. Typically weights in these models are initialized to small values early on and hence these models should be more sensitive to input early in development compared to later in the development. Knowledge learned early in L2 learning can therefore become entrenched and can inhibit later L2 learning (e.g., N. C. Ellis, 2013; A. W. Ellis & Lambon Ralph, 2000; Monner, Vatz, Morini, Hwang, & DeKeyser, 2013).

In previous versions of the dual‐path model (Chang, 2002), the output layer used a soft‐max activation function, which creates a winner‐take‐all bias, so that the model prefers to select only one word. To test whether the logistic activation function can create a human‐like L2 sensitive period, the first simulation used this activation function for the output layer and a constant learning rate throughout the training. To aid the comparisons with the human data, the model's age was represented as the number of training trials divided by 1,000 (e.g., 1 model year refers to 1,000 training trials or epochs). We applied a learning rate of 0.1 since this level allowed the model to learn Korean to an adult level within five model years.

**Figure 3:**
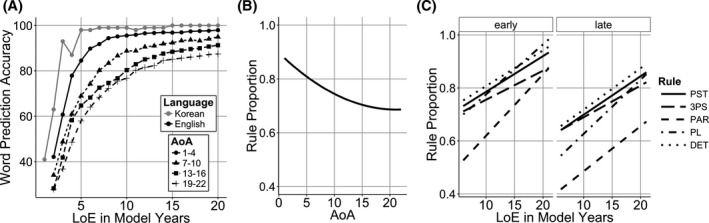
Simulation 1 model. (A) Word prediction accuracy of the Korean model (gray line) and English models that started learning English at different age of acquisition (AoA) (black lines). (B) Model rule proportion accuracy by AoA; (C) Model rule proportion by AoA, length of exposure (LoE), and Rule.

To examine the AoA effects, we looked at the overall word accuracy of the Korean models that started learning English at different AoAs. Fig. 3A shows the percentage of correctly predicted words in the Korean (gray line) and English (black lines) models that started learning the L2 at different ages. Later AoA models appeared to learn English slower, but reached similar accuracy levels after 20 model years.

To explore the model's grammatical knowledge with different rules over development, a mixed effect model was used to predict rule proportion scores with AoA, LoE, and rule CP fully crossed (Fig. 3C). All simulations contained model subject as a random intercept with random slopes for LoE crossed with Rule CP. The analysis revealed a negative effect of AoA (Fig. 3B), confirming that later AoA models performed worse than early AoA models (β = −0.01, *SE* = 0.001, χ^2^(1) = 65.8, *p* < .001). LoE effect showed that longer exposure to language resulted in better overall scores (β = 0.02, *SE* = 0.001, χ^2^(1) = 73, *p* < .001). There was a positive main effect of rule CP showing that the models performed better with the higher probability rules (β = 0.14, *SE* = 0.01, χ^2^(1) = 73.2, *p* < .001). There was a two‐way interaction between LoE and rule CP, where higher probability rule benefited more from increasing LoE (β = 0.008, *SE* = 0.002, χ^2^(1) = 16.6, *p* < .001). Finally, a three‐way interaction between AoA, LoE, and rule CP showed that this effect became stronger as AoA increased (β = 0.001, *SE* = 0.0003, χ^2^(1) = 4.07, *p* = .04).

In sum, Simulation 1 showed a negative effect of AoA and this is consistent with connectionist models where the logistic function creates an age‐dependent reduction in learning ability (A. W. Ellis & Lambon Ralph, 2000; Zevin & Seidenberg, 2002). However, the results of this model are different from those in Flege et al.'s (1999) data in several important ways (compare Fig. 1A vs. Fig. 3B). The sensitive period created by the logistic function is smaller than the one in human learners. Connectionist models learn from the input and therefore there is large LoE effect in the model. Late AoA human learners in Flege et al.'s data also showed lower sensitivity to LoE (Fig. 1B), but the present model shows no interaction between LoE and AoA (Fig. 3C). Furthermore, the human results showed a negative effect of rule CP, whereas the present model shows a positive effect. Finally, there is evidence that the sensitive period limits ultimate language attainment even with extensive input (DeKeyser & Larson‐Hall, 2005), but the present model is able to catch up with early learners and hence does not match this aspect of human learning. For example, one of the participants in Flege et al. study scored only 58% judging the grammaticality of PL rule use even after 25 years of English input (model is closer to 90% at 20 model years). So while the logistic function can create age‐dependent changes in learning, it does not capture the full behavior of L2 learners.

#### 4.4.2 Simulation 2: Stretched Z learning rate function for the sensitive period

**Figure 4:**
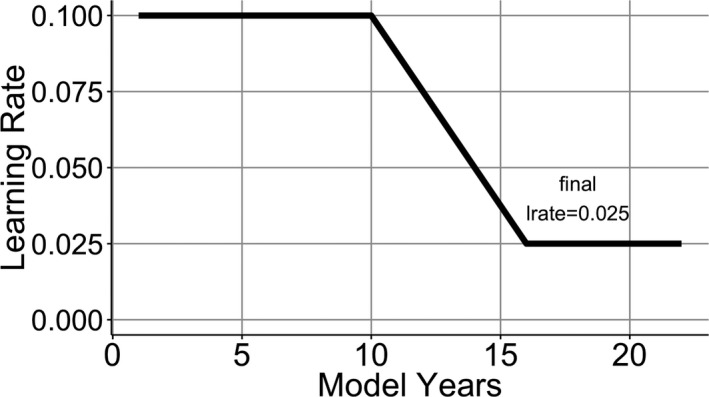
Learning rate as a function of model years.

Simulation 1 showed that activation function was not sufficient to create a human‐like sensitive period. To make the effects stronger, we directly changed the model's learning rate as it aged. There is evidence that the sensitive period has a stretched Z function (Birdsong, 2005; Flege et al., 1999; Granena & Long, 2013; Johnson & Newport, 1989; Mayberry & Eichen, 1991), where performance is high initially, but then declines gradually and is followed by a period of slower learning. These developmental changes were incorporated into the model by keeping the learning rate high (0.1) until model year 10, after which, the learning rate dropped to 0.025 over the following 6 model years (Fig. 4). With this learning rate function, later learners will have a lower learning rate in development and that might keep them from changing their Korean representations to the extent that would allow them predict English sentences with high accuracy.

Also, since the previous L1 work with the dual‐path model used the soft‐max function on the output layer (Chang, 2002), the following simulations will use that activation function to increase the similarity between the model's account of L1 and L2 learning.

**Figure 5:**
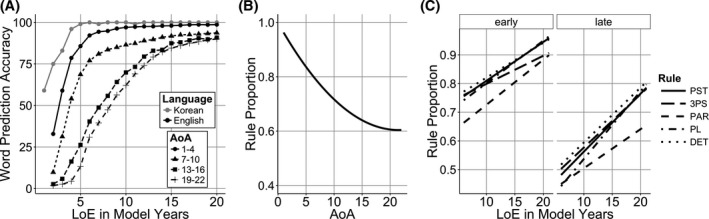
Simulation 2 model. (A) Word prediction accuracy of the Korean model (gray line) and English models that started learning English at different age of acquisition (AoA) (black lines). (B) Model rule proportion accuracy by AoA. (C) Model rule proportion by AoA, length of exposure (LoE), and Rule.

Fig. 5A shows the percentage of correctly predicted words in Korean (gray line) and English (black lines) models that started learning L2 at a different age. While all models reached high scores with enough training, the speed with which they achieved it was slower in later AoA models.

Statistical analysis confirmed that there was a significant negative effect of AoA (Fig. 5B), indicating that later AoA models had greater difficulty in distinguishing grammaticality (β = −0.02, *SE* = 0.001, χ^2^(1) = 94.4, *p* < .001). There was a positive effect of LoE (β = 0.01, *SE* = 0.001, χ^2^(1) = 101, *p* < .001), which showed that language exposure increased the models’ accuracy, and a positive effect of rule CP (β = 0.07, *SE* = 0.007, χ^2^(1) = 51.6, *p* < .001), which demonstrated that they performed better with higher CP rules (Fig. 5C). There was a positive two‐way interaction between AoA and LoE, showing that later AoA models benefited from increasing LoE more that early AoA models (β = 0.0005, *SE* = 0.0001, χ^2^(1) = 11.6, *p* < .001). There was also a positive interaction between LoE and rule CP, showing higher CP rules were more sensitive to increasing LoE than lower CP rules (β = 0.005, *SE* = 0.0001, χ^2^(1) = 20.3, *p* < .001). Finally, a three‐way interaction between AoA, LoE, and rule CP showed that this effect became stronger as AoA increased (β = 0.0005, *SE* = 0.0001, χ^2^(1) = 11.9, *p* < .001).

The reduction in the learning rate created a stronger sensitive period effect that resembles the human data more closely (compare Fig. 1A and 5B). However, like Simulation 1, the late learning models acquired the language to near native levels (Fig. 5A) and the effects of rule CP and the interaction between LoE and AoA were in the opposite direction to the corresponding effects in the human data.

#### 4.4.3 Simulation 3: Lexical and syntactic learning rates

Cognitive and neurobiological explanations of sensitive period often focus on differences between lexical and syntactic learning (Paradis, 2004; Ullman, 2015). This distinction is supported by the studies of feral children like Genie, who started learning her first language at 13 and was able to learn new words faster than other children in the same MLU stage of development, but never fully mastered English grammatical knowledge (Curtiss, Fromkin, Krashen, Rigler, & Rigler, 1974; Curtiss, Fromkin, Rigler, Rigler, & Krashen, 1975; Fromkin, Krashen, Curtiss, Rigler, & Rigler, 1974). In addition, Singleton and Lengyel (1995) have argued that there is no sensitive period for vocabulary learning in either L1 or L2 language and in some cases, L2 learners outperform native learners in word learning tasks (Kaushanskaya & Marian, 2009). There is also evidence that late learners show N400 signatures for newly learned L2 words even after only 14 h of instruction (McLaughlin, Osterhout, & Kim, 2004). Weber‐Fox and Neville (1996) found reduced syntactic P600 effects in late learners (AoA > 11) for phrase structure, but lexical N400 effects were present for both early and late learner when a word appeared in a position that was not expected in terms of meaning. These studies suggest that AoA has a greater negative impact on syntactic learning than lexical learning.

**Figure 6:**
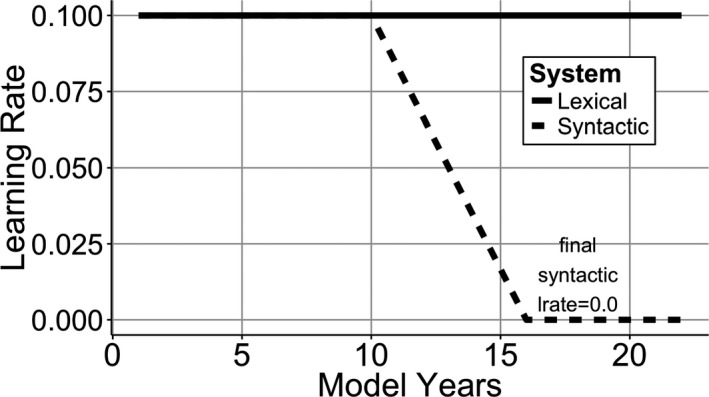
Learning rate as a function model's age in years for lexical and syntactic systems.

To examine this hypothesis in the model, we incorporated separate learning rates and varied them independently for the lexical and syntactic learning weights in the model. The lexical learning system included the connections between Concept and Produced Word layers and the connections between Hidden, Compress, and Produced Word layers (gray arrows in Fig. 2). These parts of the model were responsible for selecting the right output word, whereas the remaining parts of the model were involved in learning structural regularities (black arrows in Fig. 2). The syntactic learning rate remained fixed at 0.1 for the first 10 model years and then was reduced to 0 across the following 6 years. The learning rate in the lexical learning part of the system remained fixed at 0.1 throughout training (Fig. 6).

The focus on the distinct properties of the lexical and syntactic systems is similar to Ullman's (2001) declarative/procedural theory. In his theory, syntactic rule learning depends on implicit procedural learning and this is in agreement with our model, which only implements implicit statistical learning (Chang, Janciauskas, & Fitz, 2012). However, Ullman's theory argues that lexical learning involves declarative systems. In our model, long‐term lexical knowledge is also learned though procedural learning. The fact that procedural learning is involved in lexical learning is supported by studies showing that word‐based repetition priming is present in anterograde amnesic patients, even though their declarative learning systems are damaged (Gordon, 1988; Mayes & Gooding, 1989; Schacter & Graf, 1986). This type of priming has been argued to reflect implicit learning processes (Oppenheim, Dell, & Schwartz, 2010). However, the higher learning rate for lexical learning in the present simulation could help to support fast learning of arbitrary associations and this is one of the features of declarative memory. Thus, while this simulation has similar assumptions to Ullman's account, the model does not fully implement the declarative components of his account.

**Figure 7:**
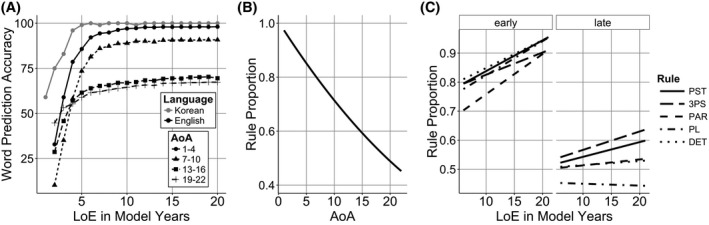
Simulation 3 model. (A) Word prediction accuracy of the Korean model (gray line) and English models that started learning English at different age of acquisition (AoA) (black lines). (B) Model rule proportion accuracy by AoA. (C) Model rule proportion by AoA, length of exposure (LoE), and Rule.

The learning rate changes in the structure learning system created a clear sensitive period effect, where later AoA models performed noticeably worse than early AoA models. However, the later AoA models were still able to use the lexical learning system to support their English grammatical knowledge and their accuracy levels approached 65% (Fig. 7A).

Analysis of rule learning revealed that there was a significant negative effect of AoA (Fig. 7B, β = −0.02, *SE* = 0.001, χ^2^(1) = 35.8, *p* < .001), a positive effect of LoE (β = 0.004, *SE* = 0.001, χ^2^(1) = 131, *p* < .001) and a marginal negative effect of rule CP (β = −0.09, *SE* = 0.01, χ^2^(1) = 3.1, *p* = .08). There were also three negative interactions between AoA and LoE (β = −0.0006, *SE* = 0.0001, χ^2^(1) = 4.87, *p* = .03), AoA and rule CP (β = −0.02, *SE* = 0.001, χ^2^(1) = 71.1, *p* < .001), and LoE and rule CP (β = −0.007, *SE* = 0.001, χ^2^(1) = 23.5, *p* < .001). Finally, there was a three‐way interaction between AoA, LoE, and rule CP (Fig. 7C), showing that with increasing AoA, higher CP rules benefited from increasing LoE less than lower CP rules (β = −0.001, *SE* = 0.0001, χ^2^(1) = 37.9, *p* < .001).

Separating lexical and syntactic learning parts of the system successfully captures the effects observed in the Flege et al. data. Importantly, it showed that the LoE effect was weaker in later AoA models (Fig. 5C). Also, the direction of the rule CP effect flipped from positive to negative. While the main effect of CP was marginal, its interaction with AoA and especially the three‐way interaction between AoA, LoE, and rule CP matched the human results showing that with increasing AoA, higher CP rules benefitted from increasing LoE less than lower CP rules.

After 16 years, the model's syntactic learning rate goes to zero and therefore the late learning models are learning to predict English words using Korean syntactic knowledge. Fig. 7A shows that 19–22 learners do acquire the ability to correctly predict English words with an accuracy of around 70%. This relates to ERP evidence showing that late L2 learners exhibit similar syntactic P600 effects as native L1 speakers in some conditions (Foucart & Frenck‐Mestre, 2011; Sabourin et al., 2006). These effects are sometimes used to argue against critical period effects, since late learners are exhibiting similar patterns to native speakers. However, even though the late learning models do not have native‐like L2 syntactic representations, their L1 representations are sufficient to create differences across L2 rules. This is especially the case when behavior across the whole network/brain is averaged into a single measure like Rule Proportion/ERPs, where it can appear as if human/model learners are processing L2 sentences in a native‐like manner.

In this and the previous simulations, the models stopped receiving Korean language input once English was introduced as an L2. Although the complete suspension of L1 input is rare, there are many L2‐dominant bilinguals (Flege, Mackay, & Piske, 2002), particularly those with early AoA with long LoE in strongly monolingual environments who would be well characterized by this model. Furthermore, there are two populations which are similar to these models in that they show AoA effects even though they mainly receive input from one language: international adoptees and deaf learners of sign language. International adoptees are adopted into a new culture and exclusively get input from one language. Several studies have found that, while these learners have similar motivation and input to native learners, they acquire the language to a lower level than the equivalent native learners and language proficiency is negatively related to age of adoption (Gardell, 1979; Gauthier & Genesee, 2011; Hyltenstam et al., 2009). Deaf learners of sign languages also show AoA effects, even though sign language is their L1 and they are highly motivated (Boudreault & Mayberry, 2006; Mayberry, 2010; Mayberry & Eichen, 1991). These AoA effects support DeKeyser and Larson‐Hall (2009, p. 88) claim that “AoA keeps playing a large role when social and environmental variables are removed” and this suggests that some biological changes in learning ability may be involved in creating the sensitive period. Although the sensitive period is evident even when learning a single language, it is the case that most L2 learners continue to use the L1 after they start to receive L2 input and we examine whether this has an effect in simulation 4.

#### 4.4.4 Simulation 4: Korean and English input in L2 learning

Our final simulation examines whether the results of the previous analyses generalize to an environment where the models receive both English and Korean input. Initially, the model learned Korean as an L1 and then it was given half‐English and half‐Korean input interleaved in a random order (akin to balanced bilinguals). To signal the target language, an additional language feature was added to the event semantics, which told the model which language it was producing. The syntactic and lexical learning rate parameters as well as other aspects of the simulation were identical to Simulation 3.

**Figure 8:**
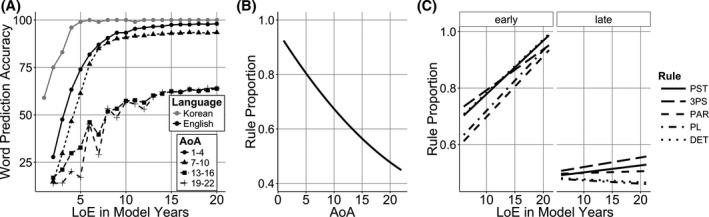
Simulation 4 model. (A) Word prediction accuracy of the Korean model (gray line) and English models that started learning English at different age of acquisition (AoA) (black lines) (B) Model rule proportion accuracy by AoA. (C) Model rule proportion by AoA, length of exposure (LoE), and Rule.

As in Simulation 3, late learning models did not achieve native‐like language accuracy (Fig. 8A). There was a negative main effect of AoA effect (Fig. 8B, β = −0.02, *SE* = 0.007, χ^2^(1) = 27.5, *p* < .001), a positive effect of LoE (β = 0.01, *SE* = 0.0004, χ^2^(1) = 137.6, *p* < .001), and a negative effect of rule CP (β = −0.08, *SE* = 0.007, χ^2^(1) = 45.8, *p* < .001). There was a negative interaction between AoA by LoE (β = −0.001, *SE* = 0.0001, χ^2^(1) = 37.3, *p* < .001), and a negative interaction between AoA and CP (β = −0.02, *SE* = 0.001, χ^2^(1) = 28.6, *p* < .001). There was also a marginal interaction between LoE by rule CP (β = −0.002, *SE* = 0.001, χ^2^(1) = 3.24, *p* = .007). Finally, there was a three‐way interaction between AoA, LoE, and rule CP (Fig. 8C, β = −0.001, *SE* = 0.0001, χ^2^(1) = 82.8, *p* < .001).

**Figure 9:**
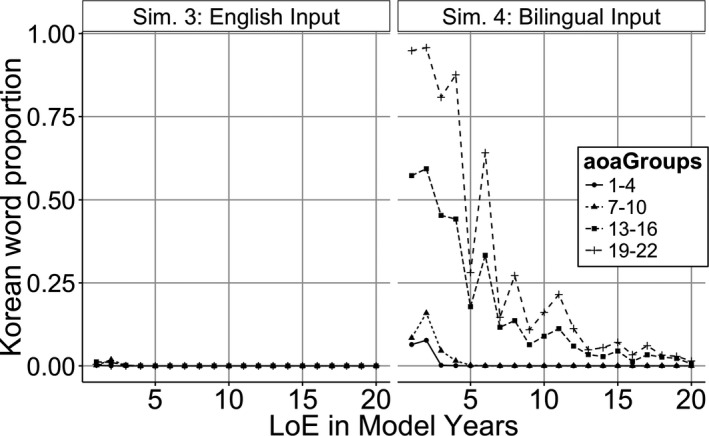
Proportion of L1 Korean words produced by English‐only model and by bilingual models at different age of acquisitions (AoAs) over length of exposure (LoE).

To better understand how bilingual input affected learning, we also examined the model's code‐switching behavior (e.g., producing Korean words in English sentences) in both simulations. Fig. 9 shows the proportion of Korean words produced by the models that received English‐only L2 training (Simulation 3) or English and Korean L2 training (Simulation 4). Late AoA models in Simulation 4 continued using many Korean words in English sentences even after a substantial number of years of English input. These results approximate the results of studies which have found that code‐switching rate was higher (14%) in late learners than in early learners (6%; Sheng, Bedore, Peña, & Fiestas, 2013). Code‐switching is very context dependent and this model does not fully capture all the factors that influence code‐switching. For example, Moore (2013) found that English‐learning Japanese speakers often switched to their L1 while preparing for an English presentation and the percentage of L1 could vary greatly within the same speaker depending on the proficiency of the interlocutor. Although AoA information was not provided for the learners in this study, there were some participants who used their L1 approximately 88% of the time, which approximates the high levels in late learners in Simulation 4.

In contrast to the marginal effect of CP in Simulation 3, the bilingual input in this simulation created a significant negative effect of CP. This means that even though the input for DET/PL was higher in the model's input, the model learned these rules less well compared to less frequent rules like 3PS/PST. We will discuss the source of these effects in the discussion. Overall, this model provided a good match to the effects of AoA, LoE, and CP seen in the Flege et al.'s reanalysis. In addition, it provided some evidence for code‐switching behavior within a model of sentence production that has learned both L1 and L2.

## 5 General discussion

This study of L2 learning examined the interaction between AoA and input factors like LoE and CP. In support of a critical/sensitive period, our reanalysis of Flege et al.'s (1999) data found a significant effect of AoA on L2 linguistic behaviors. Some studies have argued that entrenchment with connectionist activation functions can explain sensitive period effects (A. W. Ellis & Lambon Ralph, 2000; Munakata & McClelland, 2003). Simulation 1 examined this and found that these mechanisms alone were not sufficient to explain all the features of the sensitive period in the learning of grammatical knowledge. To simulate the sensitive period effects seen in humans, we changed the model's learning rates following a stretched Z function (Granena & Long, 2013). Our claim is that this learning rate is an age‐dependent learning parameter that influences L1 and L2 learning equally (some L1 phenomena can also be explained with learning rate changes, e.g., Peter, Chang, Pine, Blything, & Rowland, 2015). We can contrast this with the view that the critical period reflects specialized *linguistic* parameters, such as a head‐direction parameter (e.g., Chomsky & Lasnik, 1993), which are set within the critical period. Instead, the use of general learning parameters here suggests that linguistic critical periods could be due to mechanisms that evolved originally for non‐linguistic critical period phenomena (Knudsen, 2004; chick imprinting; Lorenz, 1937; birdsong; Marler, 1970; cochlear implants; Harrison, Gordon, & Mount, 2005).

The learning rate changes in the model may also have a role in social/motivational/input‐based accounts of the sensitive period. For example, it could be the case that children receive more optimal input for language learning than adults. In order for this input to create sensitive period effects, the knowledge that is learned from early optimal input should not be overwritten by the sometimes more than 20 years of less optimal adult input. The model's stretched Z learning function is one way to ensure that early experiences due to various factors persist in spite of further learning. Thus, regardless if one believes in a purely biological account of the sensitive period, or in a social/motivational/input‐based account, there needs to be an age‐dependent learning mechanism that insures that this early experience persists such that it can influence testing that takes place years later.

The main impetus for the present work was the finding that the amount of L2 input was a poor predictor of proficiency (DeKeyser, 2000; DeKeyser et al., 2010; Johnson & Newport, 1989; Lee & Schacter, 1997; McDonald, 2000). Such findings are compounded by evidence suggesting that some L2 learners are better at recognizing the grammatical use of lower frequency rules like the third person singular than higher frequency rules like determiners (Flege et al., 1999; Johnson & Newport, 1989). To explain this, we used corpus analyses to characterize the frequency of different rules (rule CP) and used this to factor out rule variation. When rule CP was added to the Flege et al.'s reanalysis, LoE went from non‐significant to a significant positive effect, which suggests that the lack of LoE effects in some studies may be due to the fact that this effect was obscured by rule variation. LoE was also significant when rule was included as a factor, which demonstrates that this result does not depend on a particular approach to computing rule CPs.

We also found that late AoA learners were less sensitive to the input (LoE) than early AoA learners. Our simulation 2 showed that the stretched Z learning function was not sufficient to explain this interaction. To model this effect in simulation 3, we assigned separate learning rates to the lexical and syntactic parts of the system (Paradis, 2004; Ullman, 2001). The lexical part retained a high learning rate throughout the training, whereas the syntactic learning rate followed the stretched Z function. The early AoA models had a high syntactic learning rate, which allowed them to reconfigure their Korean syntactic representations into representations that were more appropriate for English. However, the later AoA models had a low syntactic learning rate and hence their high lexical learning rate forced them to associate English words with sequence representations that were still partially Korean. On this account, the weaker effect of LoE in late AoA learners is due loss of syntactic learning ability in the late learners and their greater dependence on lexical learning as a result. This account is supported by ERP studies of L2 learners’ brain activity that have found that syntactic components such as the P600 differ from native learners more than lexical‐semantic components such as the N400 (e.g., Hahne, 2001; Hahne & Friederici, 2001; Weber‐Fox & Neville, 1996). Furthermore, recent studies have tested grammatical distinctions that yield P600 effects in native speakers and proficient L2 learners, but which yield N400 effects in some late AoA L2 learners (McLaughlin et al., 2010). Since the N400 is traditionally associated with lexical/semantic expectations, N400 effect for a grammatical distinction supports the claim that late AoA learners may be using lexical learning to a greater degree than early AoA learners to support their syntactic processing in the L2.

Although the syntactic learning rate in the model was completely switched off at age 16, this did not fully impair the model's ability to learn syntactic regularities and to differentiate between different rules. This is because the lexical and syntactic learning rates are both being used to learn word regularities that support syntactic grammaticality judgments (e.g., DET rule depends on predicting the word *the* after verbs). This means that lexical and syntactic behaviors may not be transparently related to lexical and syntactic learning in human and model behavior (see the syntactic/lexical division of labor in Chang, 2002; Gordon & Dell, 2003). For example, Granena and Long (2013) argued that lexical learning ability follows a similar negative learning function as syntactic learning, but their measure of lexical learning involves multi‐word collocations, which in our model would be encoded in the sequencing system and would be sensitive to the syntactic learning rate. We have shown here that lexical learning can be used to learn grammaticality constraints in a way that mimics the behavior in late L2 learners. Overall, our account predicts that under similar input conditions, early AoA learners can use their higher syntactic learning rate to learn deeper and more abstract syntactic rules than later AoA learners and support for this can be found in Hudson Kam and Newport (2005) study, which found that children were more likely than adults to regularize the artificial language that they were taught.

Although input is important for L2 learning, some L2 learners appear to perform worse with higher frequency rules like determiners than lower frequency rules like third‐person singular. There was a significant negative effect of rule CP in our reanalysis of Flege et al. (1999) study and similar effects have been found in other studies (DeKeyser, 2000; Johnson, 1992; McDonald, 2000; Murakami & Alexopoulou, 2016). Since the effect is negative, it is not straightforwardly explained by input‐based theories (N. C. Ellis, 2002). A likely explanation is transfer/interference from the L1, but it is often hard to formalize the morphosyntactic similarity across languages. The fact that the model does not capture this negative relationship in Simulation 1 and 2 suggests that the separate learning rates for lexical and syntactic knowledge in Simulation 3 and 4 are important in capturing these effects. Low‐frequency rules like PST and 3PS were relatively simple and the late learning models were able to correctly predict English structures with Korean syntactic representation using lexical learning to linking English words with these representations. The higher frequency DET/PL/PAR rules were more complex and harder to predict from Korean representations (these rules depend more on learned syntactic knowledge). What the model highlights is an implicit assumption of transfer accounts, which is that transfer from the L1 assumes that the L2 syntax is learned slowly enough to make it preferable to link L2 words to L1 structures and this assumption is instantiated by a gradual reduction in the syntactic learning rate, whereas lexical learning rate remained high. Although we do not know the exact nature of the L1/L2 similarity that determines transfer/interference between languages, the model provides an explicit implementation of a mechanism that captures some of these transfer effects and future work should examine the nature of this mechanism and its relation to equivalent transfer effects in human studies.

The models presented here are not fully realistic simulations of L2 learners. Rather, like the mixed model reanalysis, they provided a simplified representation of a complex pattern of data. It is also not the case that one simulation is the best simulation of all L2 speakers. It may be the case that early AoA learners and learners with greater LoE are more likely to be exposed to exclusively L2 input as in Simulation 3 (L2‐dominant bilinguals; Flege et al., 2002), whereas late AoA learners and learners who have only a short LoE are more likely to maintain connections to their L1 as in Simulation 4 (balanced bilinguals). Furthermore, different results would arise if the same model was trained on different L1/L2 pairs (Murakami & Alexopoulou, 2016) and the present simulations do not explain variation in implicit and explicit aspects of L2 tasks (R. Ellis, 2005; Chang et al., 2012). The main purpose of these models is to offer a starting point for developing a computational account of L2 learning.

The main innovation in the present work is the demonstration that a model of L1 language acquisition and production can explain L2 performance over various AoA, LoE, and grammatical rules. The extension to L2 learning involved minor changes in learning rates without any major architectural changes. Since the same network/mechanism is used for encoding L1 and L2 rules, the model predicts that there will be transfer between L1 and L2 structures (Foucart & Frenck‐Mestre, 2012; Hartsuiker et al., 2004; Ionin & Montrul, 2010; MacWhinney, 2005; Sabourin et al., 2006) and similar brain areas/ERP signatures for L1 and L2 processing (Friederici, Steinhauer, & Pfeifer, 2002; Kotz, 2009). Learning rate variation in syntactic and lexical systems offers an account which allows the same learning mechanism and network to explain the large differences due to AoA. Overall, this approach provides an explicit account of the complex interactions of various aspects of L1 and L2 structure learning.

## Appendix A

### A.1

The simulations were implemented using Version OSX‐1.0a of the LENS connectionist software package (Brouwer, de Kok, & Fitz, 2013; Rohde, 1999). Unless stated otherwise, default parameters of the simulator were used. Simulation is available at https://sites.google.com/site/sentenceproductionmodel/Home/l2model.

The model had 109 units in the Previous Word/Produced Word layers, 20 units in the Compress/CCompress layers, 30 units in the Hidden/Context layers, 5 units in the Role/CRole/CRole Copy layers, 43 units in the Concept/CConcept layers, and 6 units in the Event Semantics layer. A Context layer held a copy of the activation of the Hidden layer and was reset to 0.5 at the start of an utterance. The Previous Word layer received one‐to‐one inputs from all the Produced Word layer units and from the previous target inputs, and a winner‐take‐all filter was applied. Thus, during learning from the speech of others, the Previous Word was set to the heard target word. However, when the model was generating its own utterance, the Previous Word layer was set to the word that model had previously produced.

The CRole layer had soft‐max activation function. The CRole Copy layer (Fig. 2, top panel, center) which averaged a copy of its own previous activation with the activation of the CRole layer. To help the model learn the links between the previous word and its appropriate concept (i.e., the weights between the Previous Word and CConcept layers, Fig. 2, far left), the previous activation of the Concept layer was used as a training signal for the CConcept layer. The Role‐Concept links in the production message, the CConcept‐CRole links in the comprehension message, and Event Semantics activations were all set before a training or test sentence was processed.

Unless specified otherwise, units in all layers used the sigmoidal logistic activation function, with activation values running between 0 and 1. The Produced Word layer used the logistic activation function in simulation 1 and soft‐max in the other simulations. Weights were initially set to the values uniformly sampled between −0.5 and 0.5. Units were unbiased, unless specifically mentioned, to make the layers more dependent on their inputs for their behavior. However, Concept units were biased to −3 to ensure that they had a low default activation level.

A version of back‐propagation was used to train the model where derivatives were clipped at 1.0 (Doug's momentum; Rohde, 1999). Momentum was 0.9. Weights were updated after each message‐sentence pair had been trained. Training began by randomizing all weights (same model seed was used for all runs, but input generation used different seeds). At the start of each utterance, the message was set. After the sentence was generated, the sequence of Produced Word activations was processed by a decoder program that yielded the produced sentence. Sentences were then processed by a syntactic coder program that added the syntactic and message tags. The model's output was compared with the target sentence and the sentence was considered accurate if the all the words were correctly produced.

## References

[cogs12519-bib-0001] Ambridge, B. , Kidd, E. , Rowland, C. F. , & Theakston, A. L. (2015). The ubiquity of frequency effects in first language acquisition. Journal of Child Language, 42(02), 239–273. 25644408 10.1017/S030500091400049X PMC4531466

[cogs12519-bib-0002] Andringa, S. (2014). The use of native speaker norms in critical period hypothesis research. Studies in Second Language Acquisition, 36(03), 565–596.

[cogs12519-bib-0003] Aslin, R. N. , Saffran, J. R. , & Newport, E. L. (1998). Computation of conditional probability statistics by 8‐month‐old infants. Psychological Science, 9(4), 321–324.

[cogs12519-bib-0004] Bannard, C. , & Matthews, D. (2008). Stored word sequences in language learning: The effect of familiarity on children's repetition of four‐word combinations. Psychological Science, 19, 241–248. 18315796 10.1111/j.1467-9280.2008.02075.x

[cogs12519-bib-0005] Barr, D. J. , Levy, R. , Scheepers, C. , & Tily, H. J. (2013). Random effects structure for confirmatory hypothesis testing: Keep it maximal. Journal of Memory and Language, 68(3), 255–278. 10.1016/j.jml.2012.11.001 PMC3881361 24403724

[cogs12519-bib-0006] Bates, E. , Bretherton, I. , & Snyder, L. (1988). From first words to grammar: Individual differences and dissociable mechanisms. Cambridge, MA: Cambridge University Press.

[cogs12519-bib-0007] Bernolet, S. , Hartsuiker, R. J. , & Pickering, M. J. (2013). From language‐specific to shared syntactic representations: The influence of second language proficiency on syntactic sharing in bilinguals. Cognition, 127(3), 287–306. 23548434 10.1016/j.cognition.2013.02.005

[cogs12519-bib-0008] Bernstein‐Ratner, N. (1984). Patterns of vowel modification in motherese. Journal of Child Language, 11, 557–578. 6501465

[cogs12519-bib-0009] Birdsong, D. (2005). Interpreting age effects in second language acquisition In Kroll J. F. & de Groot A. M. B. (Eds.), Handbook of bilingualism: Psycholinguistic approaches (pp. 109–127). Oxford, UK: Oxford University Press.

[cogs12519-bib-0010] Bliss, L. (1988). The development of modals. The Journal of Applied Developmental Psychology, 9, 253–261.

[cogs12519-bib-0011] Bloom, L. (1970). Language development: Form and function in emerging grammars. Cambridge, MA: MIT Press.

[cogs12519-bib-0012] Bloom, L. (1973). One word at a time: The use of single‐word utterances before syntax. The Hague: Mouton.

[cogs12519-bib-0013] Bohannon, J. N. , & Marquis, A. L. (1977). Children's control of adult speech. Child Development, 48, 1002–1008.

[cogs12519-bib-0014] Boudreault, P. , & Mayberry, R. I. (2006). Grammatical processing in American Sign Language: Age of first‐language acquisition effects in relation to syntactic structure. Language and Cognitive Processes, 21(5), 608–635.

[cogs12519-bib-0015] Brent, M. R. , & Siskind, J. M. (2001). The role of exposure to isolated words in early vocabulary development. Cognition, 81, 31–44. 10.1016/s0010-0277(01)00122-6 11376642

[cogs12519-bib-0016] Brouwer, H. , de Kok, D. , & Fitz, H. (2013). LensOSX [Software]. Available at: http://hbrouwer.github.io/lensosx/. Accessed July 20, 2014.

[cogs12519-bib-0017] Brown, R. (1973). A first language: The early stages. Cambridge, MA: Harvard University Press.

[cogs12519-bib-0018] Bybee, J. (2006). Frequency of use and the organization of language. Oxford, UK: Oxford University Press.

[cogs12519-bib-0019] Chang, F. (2002). Symbolically speaking: A connectionist model of sentence production. Cognitive Science, 26(5), 609–651.

[cogs12519-bib-0020] Chang, F. (2009). Learning to order words: A connectionist model of heavy NP shift and accessibility effects in Japanese and English. Journal of Memory and Language, 61(3), 374–397.

[cogs12519-bib-0021] Chang, F. , Baumann, M. , Pappert, S. , & Fitz, H. (2015). Do lemmas speak German? A verb position effect in German structural priming. Cognitive Science, 39(5), 113–1130. 10.1111/cogs.12184 25307166

[cogs12519-bib-0022] Chang, F. , Dell, G. S. , & Bock, K. (2006). Becoming syntactic. Psychological Review, 113(2), 234–272. 16637761 10.1037/0033-295X.113.2.234

[cogs12519-bib-0023] Chang, F. , Janciauskas, M. , & Fitz, H. (2012). Language adaptation and learning: Getting explicit about implicit learning: Language Adaptation. Language and Linguistics Compass, 6(5), 259–278.

[cogs12519-bib-0024] Chang, F. , Lieven, E. , & Tomasello, M. (2008). Automatic evaluation of syntactic learners in typologically‐different languages. Cognitive Systems Research, 9(3), 198–213.

[cogs12519-bib-0025] Chomsky, N. A. , & Lasnik, H. (1993). The theory of principles and parameters In von Stechow J., Jacobs A., Sternefeld W., & Vennemann T. (Eds.), Syntax: An international handbook of contemporary research. Berlin: De Gruyter.

[cogs12519-bib-0027] Christiansen, M. H. , & Chater, N. (1999b). Toward a connectionist model of recursion in human linguistic performance. Cognitive Science, 23(2), 157–205.

[cogs12519-bib-0028] Clark, E. V. (1978). Awareness of language: Some evidence from what children say and do In Sinclair R. J. A. & Levelt W. (Eds.), The child's conception of language (pp. 17–43). Berlin: Springer Verlag.

[cogs12519-bib-0029] Curtiss, S. , Fromkin, V. , Krashen, S. , Rigler, D. , & Rigler, M. (1974). The linguistic development of Genie. Language, 50(3), 528–554.

[cogs12519-bib-0030] Curtiss, S. , Fromkin, V. , Rigler, D. , Rigler, M. , & Krashen, S. (1975). An update on the linguistic development of Genie In Dato D. P. (Ed.), Developmental psycholinguistics: Theory and applications. Washington, D.C.: Georgetown University Press.

[cogs12519-bib-0031] Dąbrowska, E. , & Lieven, E. (2005). Towards a lexically specific grammar of children's question constructions. Cognitive Linguistics, 16(3), 437–474.

[cogs12519-bib-0032] Davies, M. (2010). The Corpus of Contemporary American English as the first reliable monitor corpus of English. Literary and Linguistic Computing, 25(4), 447–464.

[cogs12519-bib-0033] DeKeyser, R. (2000). The robustness of critical period effects in second language acquisition. Studies in Second Language Acquisition, 22(04), 499–533.

[cogs12519-bib-0034] DeKeyser, R. , Alfi‐Shabtay, I. , & Ravid, D. (2010). Cross‐linguistic evidence for the nature of age effects in second language acquisition. Applied Psycholinguistics, 31(03), 413–438.

[cogs12519-bib-0035] DeKeyser, R. , & Larson‐Hall, J. (2005). What does the critical period really mean? In Kroll J. F. & de Groot A. M. B. (Eds.), Handbook of bilingualism: Psycholinguistic approaches (pp. 88–108). New York: Oxford University Press.

[cogs12519-bib-0036] DeKeyser, R. , & Larson‐Hall, J. (2009). What does the critical period really mean In Kroll J. F. & De Groot A. M. B. (Eds.), Handbook of bilingualism: Psycholinguistic approaches (pp. 88–108). New York: Oxford University Press.

[cogs12519-bib-0037] Demetras, M. (1989). Changes in parents’ conversational responses: A function of grammatical development. Paper presented at ASHA, St. Louis, MO.

[cogs12519-bib-0038] Ellis, N. C. (2002). Frequency effects in language processing. Studies in Second Language Acquisition, 24(02), 143–188.

[cogs12519-bib-0039] Ellis, R. (2004). The definition and measurement of L2 explicit knowledge. Language Learning, 54(2), 227–275.

[cogs12519-bib-0040] Ellis, R. (2005). Measuring implicit and explicit knowledge of a second language. Studies in Second Language Acquisition, 27(2), 141–172.

[cogs12519-bib-0041] Ellis, R. (2006). Modeling learning difficulty and second language proficiency: The differential contributions of implicit and explicit knowledge. Applied Linguistics, 27(3), 431–463.

[cogs12519-bib-0042] Ellis, N. C. (2013). Second language acquisition In Trousdale G. & Hoffmann T. (Eds.), Oxford handbook of construction grammar (pp. 365–378). Oxford, UK: Oxford University Press.

[cogs12519-bib-0043] Ellis, A. W. , & Lambon Ralph, M. A. (2000). Age of acquisition effects in adult lexical processing reflect loss of plasticity in maturing systems: Insights from connectionist networks. Journal of Experimental Psychology: Learning, Memory, and Cognition, 26(5), 1103–1123. 10.1037//0278-7393.26.5.1103 11009247

[cogs12519-bib-0044] Elman, J. L. (1993). Learning and development in neural networks: The importance of starting small. Cognition, 48, 71–99. 8403835 10.1016/0010-0277(93)90058-4

[cogs12519-bib-0045] Feldman, A. , & Menn, L. (2003). Up close and personal: A case study of the development of three English fillers. Journal of Child Language, 30(4), 735–768. 14686083 10.1017/s0305000903005774

[cogs12519-bib-0046] Fitz, H. , Chang, F. , & Christiansen, M. H. (2011). A connectionist account of the acquisition and processing of relative clauses In Kidd E. (Ed.), The acquisition of relative clauses. Vol. 8 (pp. 39–60). Amsterdam: John Benjamins.

[cogs12519-bib-0047] Flege, J. E. , Mackay, I. R. A. , & Piske, T. (2002). Assessing bilingual dominance. Applied Psycholinguistics, 23(4), 567–598.

[cogs12519-bib-0048] Flege, J. E. , Yeni‐Komshian, G. H. , & Liu, S. (1999). Age constraints on second‐language acquisition. Journal of Memory and Language, 41(1), 78–104.

[cogs12519-bib-0049] Foucart, A. , & Frenck‐Mestre, C. (2011). Grammatical gender processing in L2: Electrophysiological evidence of the effect of L1–L2 syntactic similarity. Bilingualism: Language and Cognition, 14(03), 379–399.

[cogs12519-bib-0050] Foucart, A. , & Frenck‐Mestre, C. (2012). Can late L2 learners acquire new grammatical features? Evidence from ERPs and eye‐tracking. Journal of Memory and Language, 66(1), 226–248.

[cogs12519-bib-0051] French, R. M. , Addyman, C. , & Mareschal, D. (2011). TRACX: A recognition‐based connectionist framework for sequence segmentation and chunk extraction. Psychological Review, 118(4), 614–636. 22003842 10.1037/a0025255

[cogs12519-bib-0052] Friederici, A. D. , Steinhauer, K. , & Pfeifer, E. (2002). Brain signatures of artificial language processing: Evidence challenging the critical period hypothesis. Proceedings of the National Academy of Sciences, 99(1), 529–534. 10.1073/pnas.012611199 PMC117594 11773629

[cogs12519-bib-0053] Fromkin, V. , Krashen, S. , Curtiss, S. , Rigler, D. , & Rigler, M. (1974). The development of language in Genie: A case of language acquisition beyond the “critical period.” Brain and Language, 1, 81–107.

[cogs12519-bib-0054] Gardell, I. (1979). A Swedish study of intercountry adoptions: A report from Allmänna Barnhuset. Stockholm: Allmänna Barnhuset.

[cogs12519-bib-0055] Gauthier, K. , & Genesee, F. (2011). Language development in internationally adopted children: A special case of early second language learning: Language development. Child Development, 82(3), 887–901. 21413938 10.1111/j.1467-8624.2011.01578.x

[cogs12519-bib-0056] Gleason, J. B. (1980). The acquisition of social speech and politeness formulae In Giles H., Robinson W. P., & Smith P. (Eds.), Language: Social psychological perspectives (pp. 21–27). Oxford, UK: Pergamon.

[cogs12519-bib-0057] Gomez, R. L. , & Gerken, L. (2000). Infant artificial language learning and language acquisition. Trends in Cognitive Sciences, 4(5), 178–186. 10782103 10.1016/s1364-6613(00)01467-4

[cogs12519-bib-0058] Gordon, B. (1988). Preserved learning for novel information in amnesia: Evidence for multiple memory systems. Brain and Cognition, 7, 257–282. 3401382 10.1016/0278-2626(88)90002-4

[cogs12519-bib-0501] Gordon, J. K. , & Dell, G. S. (2003). Learning to divide the labor: An account of deficits in light and heavy verb production. Cognitive Science, 27(1), 1–40. 12030471

[cogs12519-bib-0059] Granena, G. , & Long, M. H. (2013). Age of onset, length of residence, language aptitude, and ultimate L2 attainment in three linguistic domains. Second Language Research, 29(3), 311–343.

[cogs12519-bib-0060] Hahne, A. (2001). What's different in second‐language processing? Evidence from event‐related brain potentials. Journal of Psycholinguistic Research, 30(3), 251–266. 11523274 10.1023/a:1010490917575

[cogs12519-bib-0061] Hahne, A. , & Friederici, A. D. (2001). Processing a second language: Late learners’ comprehension mechanisms as revealed by event‐related brain potentials. Bilingualism: Language and Cognition, 4(2), 123–141.

[cogs12519-bib-0062] Hall, W. S. , Nagy, W. E. , & Linn, R. (1984). Spoken words: Effects of situation and social group on oral word usage and frequency. Hillsdale, NJ: Erlbaum.

[cogs12519-bib-0063] Harrison, R. V. , Gordon, K. A. , & Mount, R. J. (2005). Is there a critical period for cochlear implantation in congenitally deaf children? Analyses of hearing and speech perception performance after implantation. Developmental Psychobiology, 46(3), 252–261. 15772969 10.1002/dev.20052

[cogs12519-bib-0064] Hartsuiker, R. J. , & Pickering, M. J. (2008). Language integration in bilingual sentence production. Acta Psychologica, 128(3), 479–489. 17870040 10.1016/j.actpsy.2007.08.005

[cogs12519-bib-0065] Hartsuiker, R. J. , Pickering, M. J. , & Veltkamp, E. (2004). Is syntax separate or shared between languages? Cross‐linguistic syntactic priming in Spanish‐English bilinguals. Psychological Science, 15(6), 409–414. 15147495 10.1111/j.0956-7976.2004.00693.x

[cogs12519-bib-0066] Higginson, R. P. (1985). Fixing‐assimilation in language acquisition (Unpublished doctoral dissertation). Washington State University.

[cogs12519-bib-0067] Huang, P. Y. , Wible, D. , & Ko, H. W. (2012). Frequency effects and transitional probabilities in L1 and L2 speakers’ processing of multiword expressions In Gries S. T. & Divjak D. (Eds.), Frequency effects in language learning and processing (pp. 145–175). Berlin, Germany: Mouton de Gruyter.

[cogs12519-bib-0068] Hudson Kam, C. L. , & Newport, E. L. (2005). Regularizing unpredictable variation: The roles of adult and child learners in language formation and change. Language Learning and Development, 1(2), 151–195.

[cogs12519-bib-0069] Huettig, F. , & Mani, N. (2016). Is prediction necessary to understand language? Probably not. Language, Cognition and Neuroscience, 31(1), 19–31.

[cogs12519-bib-0070] Hyltenstam, K. , Bylund, E. , Abrahamsson, N. , & Park, H. (2009). Dominant‐language replacement: The case of international adoptees. Bilingualism: Language and Cognition, 12(02), 121–140.

[cogs12519-bib-0071] Ionin, T. , & Montrul, S. (2010). The role of L1 transfer in the interpretation of articles with definite plurals in L2 English. Language Learning, 60(4), 877–925.

[cogs12519-bib-0072] Johnson, J. S. (1992). Critical period effects in second language acquisition: The effect of written versus auditory materials on the assessment of grammatical competence. Language Learning, 42(2), 217–248.

[cogs12519-bib-0073] Johnson, P. C. D. (2014). Extension of Nakagawa & Schielzeth's R 2 GLMM to random slopes models. Methods in Ecology and Evolution, 5(9), 944–946. 25810896 10.1111/2041-210X.12225 PMC4368045

[cogs12519-bib-0074] Johnson, J. S. , & Newport, E. L. (1989). Critical period effects in second language learning: The influence of maturational state on the acquisition of English as a second language. Cognitive Psychology, 21(1), 60–99. 2920538 10.1016/0010-0285(89)90003-0

[cogs12519-bib-0075] Jurafsky, D. (2003). Probabilistic modeling in psycholinguistics: Linguistic comprehension and production In Bod R., Hay J., & Jannedy S. (Eds.), Probabilistic linguistics, (pp. 39–95). Cambridge, MA: The MIT Press.

[cogs12519-bib-0076] Kaushanskaya, M. , & Marian, V. (2009). The bilingual advantage in novel word learning. Psychonomic Bulletin & Review, 16(4), 705–710. 19648456 10.3758/PBR.16.4.705

[cogs12519-bib-0077] Knudsen, E. (2004). Sensitive periods in the development of the brain and behavior. Journal of Cognitive Neuroscience, 16(8), 1412–1425. 15509387 10.1162/0898929042304796

[cogs12519-bib-0078] Kotz, S. A. (2009). A critical review of ERP and fMRI evidence on L2 syntactic processing. Brain and Language, 109(2–3), 68–74. 18657314 10.1016/j.bandl.2008.06.002

[cogs12519-bib-0079] Kuczaj, S. (1977). The acquisition of regular and irregular past tense forms. Journal of Verbal Learning and Verbal Behavior, 16, 589–600.

[cogs12519-bib-0080] Lee, D. , & Schacter, J. (1997). Sensitive period effects in binding theory. Language Acquisition, 6, 333–362.

[cogs12519-bib-0081] Lenneberg, E. H. (1967). Biological foundations of language. New York: Wiley.

[cogs12519-bib-0082] Leonard, L. B. , Caselli, M. C. , Bartolini, U. , McGregor, K. K. , & Sabbadini, L. (1992). Morphological deficits in children with specific language impairment: The status of features in the underlying grammar. Language Acquisition, 2(2), 151–179.

[cogs12519-bib-0083] Levy, R. (2008). Expectation‐based syntactic comprehension. Cognition, 106(3), 1126–1177. 17662975 10.1016/j.cognition.2007.05.006

[cogs12519-bib-0084] Lieven, E. (2010). Input and first language acquisition: Evaluating the role of frequency. Lingua, 120(11), 2546–2556.

[cogs12519-bib-0085] Lorenz, K. (1937). The Companion in the Bird's World. The Auk, 54(1), 245–273.

[cogs12519-bib-0086] MacWhinney, B. (2000). The CHILDES project: Tools for analyzing talk (3rd ed.). Mahwah, NJ: Lawrence Erlbaum Associates.

[cogs12519-bib-0087] MacWhinney, B. (2005). A unified model of language acquisition In Kroll J. F. & de Groot A. M. B. (Eds.), Handbook of bilingualism: Psycholinguistic approaches (pp. 49–67). New York: Oxford University Press.

[cogs12519-bib-0088] MacWhinney, B. (2008). A unified model In Ellis N. C. & Robinson P. (Eds.), Handbook of cognitive linguistics and second language acquisition (pp. 341–371). New York: Routledge.

[cogs12519-bib-0089] Marchman, V. A. (1993). Constraints on plasticity in a connectionist model of the English past tense. Journal of Cognitive Neuroscience, 5(2), 215–234. 23972155 10.1162/jocn.1993.5.2.215

[cogs12519-bib-0090] Marchman, V. A. , Wulfeck, B. , & Weismer, S. E. (1999). Morphological productivity in children with normal language and SLI: A study of the English past tense. Journal of Speech, Language, and Hearing Research, 42, 206–219. 10.1044/jslhr.4201.206 10025555

[cogs12519-bib-0091] Marler, P. (1970). A comparative approach to vocal learning: Song development in white‐crowned sparrows. Journal of Comparative and Physiological Psychology, 71(2p2), 1.

[cogs12519-bib-0092] Mayberry, R. I. (2010). Early language acquisition and adult language ability: What sign language reveals about the critical. The Oxford Handbook of Deaf Studies, Language, and Education, 2, 281–291.

[cogs12519-bib-0093] Mayberry, R. I. , & Eichen, E. B. (1991). The long‐lasting advantage of learning sign language in childhood: Another look at the critical period for language acquisition. Journal of Memory and Language, 30, 486–512.

[cogs12519-bib-0094] Mayes, A. R. , & Gooding, P. (1989). Enhancement of word competition priming in amnesics by cueing with previously novel associates. Neuropsychologia, 27(8), 1057–1072. 2797413 10.1016/0028-3932(89)90185-1

[cogs12519-bib-0095] McDonald, J. L. (2000). Grammaticality judgments in a second language: Influences of age of acquisition and native language. Applied Psycholinguistics, 21, 395–423.

[cogs12519-bib-0096] McLaughlin, J. , Osterhout, L. , & Kim, A. (2004). Neural correlates of second‐language word learning: Minimal instruction produces rapid change. Nature Neuroscience, 7(7), 703–704. 15195094 10.1038/nn1264

[cogs12519-bib-0097] McLaughlin, J. , Tanner, D. , Pitkänen, I. , Frenck‐Mestre, C. , Inoue, K. , Valentine, G. , & Osterhout, L. (2010). Brain potentials reveal discrete stages of L2 grammatical learning: Brain potentials and L2 grammatical learning. Language Learning, 60, 123–150.

[cogs12519-bib-0098] Mermillod, M. , Bonin, P. , Méot, A. , Ferrand, L. , & Paindavoine, M. (2012). Computational evidence that frequency trajectory theory does not oppose but emerges from age‐of‐acquisition theory. Cognitive Science, 36(8), 1499–1531. 22985438 10.1111/j.1551-6709.2012.01266.x

[cogs12519-bib-0099] Mizumoto, T. , Hayashibe, Y. , Komachi, M. , Nagata, M. , & Matsumoto, Y. (2012). The effect of learner corpus size in grammatical error correction of ESL writings. In 24th International Conference on Computational Linguistics (p. 863–872).

[cogs12519-bib-0100] Monaghan, P. , Chater, N. , & Christiansen, M. H. (2005). The differential role of phonological and distributional cues in grammatical categorisation. Cognition, 96(2), 143–182. 15925574 10.1016/j.cognition.2004.09.001

[cogs12519-bib-0101] Monner, D. , Vatz, K. , Morini, G. , Hwang, S. , & DeKeyser, R. (2013). A neural network model of the effects of entrenchment and memory development on grammatical gender learning. Bilingualism: Language and Cognition, 16(02), 246–265.

[cogs12519-bib-0102] Moore, P. J. (2013). An emergent perspective on the use of the first language in the english‐as‐a‐foreign‐language classroom: An emergent perspective on the use of the first language. The Modern Language Journal, 97(1), 239–253.

[cogs12519-bib-0103] Morisset, C. E. , Barnard, K. E. , & Booth, C. L. (1995). Toddlers’ language development: Sex differences within social risk. Developmental Psychology, 31(5), 851–865.

[cogs12519-bib-0104] Munakata, Y. , & McClelland, J. L. (2003). Connectionist models of development. Developmental Science, 6(4), 413–429.

[cogs12519-bib-0105] Murakami, A. , & Alexopoulou, T. (2016). L1 influence on the acquisition order of English grammatical morphemes. Studies in Second Language Acquisition, 38(3), 365–401.

[cogs12519-bib-0106] Nakagawa, S. , & Schielzeth, H. (2013). A general and simple method for obtaining *R* ^2^ from generalized linear mixed‐effects models. Methods in Ecology and Evolution, 4(2), 133–142.

[cogs12519-bib-0107] Ninio, A. , Snow, C. , Pan, B. , & Rollins, P. (1994). Classifying communicative acts in children's interactions. Journal of Communications Disorders, 27, 157–188. 10.1016/0021-9924(94)90039-6 7929878

[cogs12519-bib-0108] Oppenheim, G. M. , Dell, G. S. , & Schwartz, M. F. (2010). The dark side of incremental learning: A model of cumulative semantic interference during lexical access in speech production. Cognition, 114(2), 227–252. 19854436 10.1016/j.cognition.2009.09.007 PMC2924492

[cogs12519-bib-0109] Oyama, S. (1978). The sensitive period in comprehension of speech. NABE Journal, 3(1), 25–39.

[cogs12519-bib-0110] Paradis, M. (2004). A neurolinguistic theory of bilingualism. Amsterdam: John Benjamins.

[cogs12519-bib-0111] Patkowski, M. S. (1980). The sensitive period for the acquisition of syntax in a second language. Language Learning, 30(2), 449–468.

[cogs12519-bib-0112] Peter, M. , Chang, F. , Pine, J. M. , Blything, R. , & Rowland, C. F. (2015). When and how do children develop knowledge of verb argument structure? Evidence from verb bias effects in a structural priming task. Journal of Memory and Language, 81, 1–15.

[cogs12519-bib-0113] Peters, A. (1987). The role of imitation in the developing syntax of a blind child. Text‐Interdisciplinary Journal for the Study of Discourse, 7, 289–311.

[cogs12519-bib-0114] Phillips, B. (2006). Word frequency and lexical diffusion. New York: Palgrave Macmillan.

[cogs12519-bib-0115] Post, K. (1994). Negative evidence In Sokolov J. & Snow C. (Eds.), Handbook of research in language development using CHILDES (pp. 132–173). Hillsdale, NJ: Lawrence Erlbaum Associates.

[cogs12519-bib-0116] Rescorla, L. , & Reberts, J. (2002). Nominal versus verbal morpheme use in late talkers at ages 3 and 4. Journal of Speech, Language, and Hearing Research, 45, 1219–1231. 10.1044/1092-4388(2002/098) 12546489

[cogs12519-bib-0117] Rohde, D. L. T. (1999). LENS: The light, efficient network simulator. Technical Report CMU‐CS‐99‐164, Carnegie Mellon University, Department of Computer Science, Pittsburgh, PA.

[cogs12519-bib-0118] Rollins, P. R. (2003). Caregiver contingent comments and subsequent vocabulary Comprehension. Applied Psycholinguistics, 24, 221–234.

[cogs12519-bib-0119] Rumelhart, D. E. , Hinton, G. E. , & Williams, R. J. (1986). Learning representations by back‐propagating errors. Nature, 323(9), 533–536.

[cogs12519-bib-0120] Sabourin, L. , Stowe, L. A. , & de Haan, G. J. (2006). Transfer effects in learning a second language grammatical gender system. Second Language Research, 22(1), 1–29.

[cogs12519-bib-0121] Sachs, J. (1983). Talking about the there and then: The emergence of displaced reference in parent‐child discourse In Nelson K. E. (Ed.), Children's language. Vol. 4 (pp. 1–28). New York: Gardner Press.

[cogs12519-bib-0122] Schacter, D. L. , & Graf, P. (1986). Preserved learning in amnesic patients: Perspectives from research on direct priming. Journal of Clinical and Experimental Neuropsychology, 8(6), 727–743. 3782452 10.1080/01688638608405192

[cogs12519-bib-0123] Schoonbaert, S. , Hartsuiker, R. J. , & Pickering, M. J. (2007). The representation of lexical and syntactic information in bilinguals: Evidence from syntactic priming. Journal of Memory and Language, 56(2), 153–171.

[cogs12519-bib-0124] Sheng, L. , Bedore, L. M. , Peña, E. D. , & Fiestas, C. (2013). Semantic development in Spanish‐English bilingual children: Effects of age and language experience. Child Development, 84(3), 1034–1045. 23163772 10.1111/cdev.12015 PMC3582743

[cogs12519-bib-0125] Singleton, D. , & Lengyel, Z. (1995). The age factor in second language acquisition: A critical look at the critical period hypothesis. Clevedon: Multilingual Matter.

[cogs12519-bib-0126] Soderstrom, M. , Blossom, M. , Foygel, R. , & Morgan, J. L. (2008). Acoustical cues and grammatical units in speech to two preverbal infants. Journal of Child Language, 35, 869–902. 18838016 10.1017/S0305000908008763

[cogs12519-bib-0127] Suppes, P. (1974). The semantics of children's language. American Psychologist, 29, 103–114.

[cogs12519-bib-0128] Thompson, S. P. , & Newport, E. L. (2007). Statistical learning of syntax: The role of transitional probability. Language Learning and Development, 3(1), 1–42.

[cogs12519-bib-0129] Twomey, K. E. , Chang, F. , & Ambridge, B. (2014). Do as I say, not as I do: A lexical distributional account of English locative verb class acquisition. Cognitive Psychology, 73, 41–71. 24956024 10.1016/j.cogpsych.2014.05.001

[cogs12519-bib-0130] Ullman, M. T. (2001). The neural basis of lexicon and grammar in first and second language: The declarative/procedural model. Bilingualism: Language and Cognition, 4(1), 105–122.

[cogs12519-bib-0131] Ullman, M. T. (2015). The declarative/procedural model: A neurobiologically motivated theory of first and second language In VanPatten B. & Williams J. (Eds.), Theories in second language acquisition: An introduction (2nd ed) (pp. 135–158). New York: Routledge.

[cogs12519-bib-0132] Valian, V. (1991). Syntactic subjects in the early speech of American and Italian children. Cognition, 40, 21–81. 1786672 10.1016/0010-0277(91)90046-7

[cogs12519-bib-0133] van Houten, L. (1986). Role of maternal input in the acquisition process: The communicative strategies of adolescent and older mothers with their language learning children. Paper presented at the Boston University Conference on Language Development, Boston.

[cogs12519-bib-0134] Warren‐Leubecker, A. (1982). Sex differences in speech to children (Unpublished doctoral dissertation). Georgia Institute of Technology.

[cogs12519-bib-0135] Weber‐Fox, C. , & Neville, H. (1996). Maturational constraints on functional specializations for language processing: ERP and behavioral evidence in bilingual speakers. Journal of Cognitive Neuroscience, 8(3), 231–256. 23968150 10.1162/jocn.1996.8.3.231

[cogs12519-bib-0136] Zevin, J. D. , & Seidenberg, M. S. (2002). Age of acquisition effects in word reading and other tasks. Journal of Memory and Language, 47, 1–29.

